# Ecological River Health Assessment Using Multi-Metric Models in an Asian Temperate Region with Land Use/Land Cover as the Primary Factor Regulating Nutrients, Organic Matter, and Fish Composition

**DOI:** 10.3390/ijerph19159305

**Published:** 2022-07-29

**Authors:** Md Mamun, Namsrai Jargal, Usman Atique, Kwang-Guk An

**Affiliations:** Department of Bioscience and Biotechnology, Chungnam National University, Daejeon 34134, Korea; mamun1006001@gmail.com (M.M.); jargal.namsrai.sci@gmail.com (N.J.); physioatique@gmail.com (U.A.)

**Keywords:** multi-metric fish model, river health, Asian monsoon, nutrient enrichment, fish indicator

## Abstract

This study was performed to determine the ecological health of a temperate river over nine years (2011–2019); it also analyzed the trophic structure and linkage of nutrients (nitrogen [N] and phosphorus [P]), sestonic chlorophyll-a (CHL-a), and the top trophic fish in the Asian monsoon region. Water chemistry, trophic indicators, and tolerance guilds were primarily influenced by land use and land cover (LULC); the magnitude of variation was also related to geographic elevation, artificial physical barriers (weirs), and point sources. Levels of nutrients, organic matter, and CHL-a largely influenced by the intensity of the monsoon seasonality for a particular LULC and stream order. Mann–Kendall tests based on a long-term annual dataset showed that annual organic matter and CHL-a increased over time because of longer hydraulic residence time after weir construction. The results of empirical nutrient models suggested that P was the key determinant for algal growth (CHL-a); the strong P-limitation was supported by N:P ratios > 17 in ambient waters. Linear regression models and canonical correspondence analysis (CCA) were used to determine the influences of LULC and water quality on the trophic/tolerance linkages, fish community compositions and structures, and river health. Tolerant species had a positive functional relationship with nutrient enrichment through total phosphorus (TP) (R^2^ = 0.55, *p* < 0.05) and total nitrogen (TN) (R^2^ = 0.57, *p* < 0.05), organic pollution in terms of biological oxygen demand (BOD) (R^2^ = 0.41, *p* < 0.05) and chemical oxygen demand (COD) (R^2^ = 0.49, *p* < 0.05), and algal growth (R^2^ = 0.47, *p* < 0.05); sensitive species exhibited the opposite pattern. The degradation of river health, based on the multi-metric index of biotic integrity (IBI) model, was evident in the downriver region (“fair–poor” condition) and was supported by the quantitative fish community index (QFCI) model. The outcomes suggested that the degradation and variation of ecological river health, trophic linkages of water chemistry (N, P)-algal biomass-fish, were largely controlled by the land use pattern and construction of physical barriers in relation to the Asian monsoon.

## 1. Introduction

Aquatic ecosystem health is a hot research topic in freshwater ecology [1,2]. River ecologists have attempted to assess the impacts of multiple stressors (e.g., nutrient levels, toxic contaminants, and habitat alteration) that could impair the inherent structure and functions of riverine ecosystems [1,2,3,4]. This approach is widely used for protecting (and chemically and physically restoring) degraded river systems and for assessing the current conditions of ecological river health [5]. Rapid urban expansion and industrial developments worldwide, as well as intensive farming, have contributed to the deterioration of chemical water quality and alterations of physical habitats in rivers and streams [6,7]. Such modifications influence the aquatic biota composition and trophic interactions throughout the food chain, thereby damaging the overall river health and shifting the community structures of low- to high-level trophic biota [8,9]. Concomitant modifications are more intricate in riverine ecosystems, particularly in Asian temperate river systems that are primarily regulated by the seasonal monsoon [10].

Regional seasonality directly governs river health and trophic conditions, especially in the Asian monsoon region. Previous studies have shown that Asian lotic systems are significantly impacted by the monsoon-induced flow, which has a substantial effect on fish composition [11,12]. During the monsoon, rapid river run-off reduces the water residence time (WRT, which describes how long the water stays in an aquatic system before leaving), thus influencing nutrient levels, organic matter, and light availability, which directly controls the sestonic chlorophyll-a (CHL-a) productivity [5]. Similarly, the monsoon-induced flow can be a critical determinant of fish community relocation; it changes functional relationships among nutrients, organic matter, and biological factors [5,11].

To identify such factors when assessing aquatic habitats, there is a need to develop more robust techniques and indicators of ecosystem health monitoring [13]. Consequently, many indicators and methods have been developed to assess the ecological health of river ecosystems worldwide, such as in the United States [4,14], United Kingdom [15,16], Canada [17,18], France [16], Germany [15,19], Sweden [20], Australia [21], New Zealand [22], Africa [23], Namibia [7], Sri Lanka [24], China [3], and South Korea [10]. Traditionally, river health monitoring has been based on water chemistry assessments [25]; however, recent investigations have revealed that this approach does not yield critical information about a river’s ecological health status [26]. Therefore, innovative techniques (e.g., integrated methodologies) were developed by incorporating physical, chemical, and biological processes. Such integrated approaches provide a multifaceted snapshot of the ecological health statuses of rivers and other water bodies [2,8].

Assessment methods that integrate water chemistry and biological communities have become widely used for river health assessments worldwide [3,5,20,23]. One of these assessment techniques is the water pollution index (WPI), which is used to assess chemical water quality. Other techniques include the index of biotic integrity (IBI) and the quantitative community index, which evaluate the biological community structure and tolerance score, respectively. Bach (1980) [27] introduced the WPI for assessment of chemical health in rivers; Kim and An (2015) adapted this index for analyses of nutrient, organic, ionic, and algal pollution on a regional scale. IBI models can detect alterations in an aquatic ecosystem that are manifested through shifts in the structural composition of the species present and their richness in connection with various ecological stressors [4,16]. They can also identify biological health problems associated with physical habitats, hazardous substances, and biological agents [2]. Biological health of the river indicates high taxa richness and taxonomic composition, good ecological health and sustainable ecological processes and evolutionary process [28]. Periphyton, macroinvertebrates, and fishes can be used as indicators in biological health assessments of freshwater systems [4,14,16,29].

Stark (1985) [30] first proposed the quantitative community index based on an assessment of macroinvertebrates, with the intention of recording the health statuses of streams and rivers. Fish assemblages have been used to estimate the biological health statuses of rivers in the United States [4,18], Europe [15,16,19], Oceania [21], Africa [23], and Asia [13,31]. These studies have demonstrated that fish are the most reliable bioindicators for assessing river health because of factors such as ease of sampling, availability of established taxonomic identification methods, and ability to reflect various influences (e.g., nutrient levels, organic and algal pollution, and habitat degradation) [4].

Empirical regression models based on trophic state parameters (e.g., total phosphorus [TP], total nitrogen [TN], and CHL-a) have been less extensively studied in riverine ecosystems because of the considerable variability and difficulty involved in characterizing nutrient loads, optical water quality, and trends of primary productivity [32]. Within the trophic dynamics paradigm, empirical nutrient models linking fish trophic and tolerance guilds with ecological health assessments in rivers are not often studied [2]. The primary element that affects the nutrient levels and water clarity in riverine habitats is river flow or WRT, which is directly related to the quantities of algal chlorophyll, periphyton, and macroinvertebrates; it is also related to fish composition [2,33]. Thus, the impacts of light availability and nutrient limitations on algal growth must be quantified to understand trophic interactions in the aquatic food chain at distinct trophic levels.

Trophic preferences and relationships can influence river water chemistry, community patterns, and ecological integrity. Therefore, a quantitative trophic evaluation of each taxon is needed to elucidate the functional alterations and trophic dynamics in aquatic ecosystems [34]. Despite the seasonal and temporal variability, river studies performed with a bottom-up approach have demonstrated that nitrogen (N) and phosphorus (P) levels are typically related to algal biomass [8,35]. Nutrient enrichment can alter phytoplankton biomass, species composition, and trophic interactions at various stages of the food chain [1,33,36]. The ambiguity in food chain interactions and inconsistencies in scientific data have led the authors of several studies to conclude that nutrient flows in rivers may not be directly connected to the higher trophic-level composition and biomass [37,38]. Previous studies have shown that land use and land cover (LULC) and weir construction in a watershed influence the nutrient levels, organic content, and trophic and tolerance guilds [8,39]. Furthermore, elevation has large impacts on both water quality and fish composition [2]. The LULC and presence of weirs can modify river ecosystem function by altering the WRT, water flow and volume, sediments, nutrients, and organic matter inputs; these changes substantially influence overall river health [5,40].

As one of the four major rivers in Korea, the Geum River is a tourist attraction that also provides drinking, irrigation, and industrial water, as well as habitats for fish and other aquatic life [41]. It has been damaged by rapid population expansion, intensive land use, and constant development [40]. Three weirs were constructed in the Geum River’s mainstream from 2009 to 2012 as part of the Four Major Rivers Restoration Project; these weirs have altered the function and chemical and biological component of the river’s ecosystems [5,40]. Previous studies of the Geum River revealed severe environmental health issues, such as increases in algal blooms, silt accumulation, and fish fatalities [41]. Resolution of these issues requires an understanding of cause-and-effect interactions that involve the monsoon, LULC, elevation, nutrients, organic matter, algal chlorophyll, fish tolerance, trophic guilds, and ecological health.

This study analyzed the trophic interactions of nutrients with sestonic chlorophyll. Furthermore, it investigated the fish trophic and tolerance guilds connected with the monsoon, LULC, elevation, and water chemistry. It also evaluated ecological river health by using the multi-metric indices of WPI and IBI, along with assessments that involved the quantitative fish community index (QFCI) model. These integrative evaluations could be synchronized because they provide critical information for assessing the health of rivers in temperate zones; such information can be used in various biotic indices to identify relationships among nutrients, algal chlorophyll, and higher trophic levels.

## 2. Materials and Methods

### 2.1. Study Area

**Figure 1:**
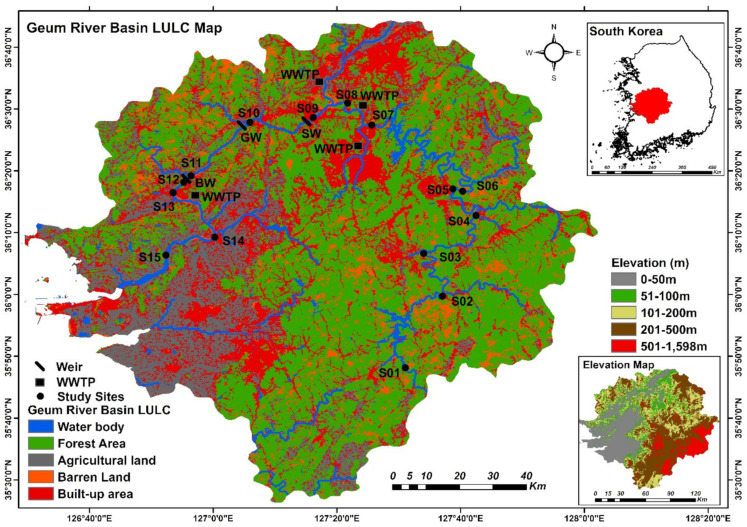
Map showing the sampling sites in the mainstream of the Geum River. SW, Sejong Weir; GW, Gongju Weir; BW, Baekje Weir; WWTP, wastewater treatment plant.

The Geum River is Korea’s third-largest river system and flows into the Yellow Sea. It is 401 km long and has a basin area of 9866 km^2^ [41]. The upper portion of the watershed is dominated by forest land; the river passes through the cities of Jinan, Geumsan, Yeongdong, and Okcheon. The route then travels through the Daejeon Metropolitan Area and the cities of Sejong, Gongju, and Buyeo, which are the major suppliers of nutrients and organic pollutants. Subsequently, it runs through Nonsan and Iksan, which are also responsible for nutrient inputs and organic loads from rice fields and livestock farming (Figure 1). Urbanization and intensive farming have had adverse effects on ecological integrity in the Geum River. The Sejong, Gongju, and Baekje weirs were constructed in the Geum River as part of the Four Major Rivers Restoration Project to regulate floods and restore environmentally damaged regions [40]. However, the river experienced catastrophic algal blooms immediately after the weirs were built. Many Korean scientists and environmentalists believe that the weirs are responsible for the destruction of ecological integrity [42]. The study area is characterized by a distinct temperate climate based on four seasons (spring, summer, fall, and winter). Approximately 60% of the total precipitation is received in the summer. The stream order information was extracted from the HydroRIVERS dataset (Lehner and Grill, 2013) and a map of the sampling sites is shown in the Supplementary file (Figure S1).

### 2.2. Water Quality Assessment, LULC and Elevation

The Korean Ministry of Environment (MOE) is authorized to collect monthly surface water quality data nationwide to quantify ecological health assessments. We obtained monthly water quality data from the MOE’s Water Information Network (http://water.nier.go.kr, accessed on 25 January 2022). This study evaluated nine water quality variables at 15 sites along the Geum River’s mainstream, from 2011 to 2019. The sampling time of water quality data was 11 ± 0.30 Am. A portable YSI Sonde Model 6600 multiparameter analyzer was utilized on-site to directly measure water temperature (WT), electrical conductivity (EC), and dissolved oxygen (DO). In addition, samples were collected, preserved, and analyzed via MOE-approved methodologies to determine total suspended solids (TSS), chemical oxygen demand (COD), biological oxygen demand (BOD), TP, TN, and CHL-a [43]. ESRI global land cover data were used to obtain LULC data for the study area [44] (https://www.arcgis.com/home/item.html?id=d3da5dd386d140cf93fc9ecbf8da5e31, accessed on 25 January 2022). This layer displays a global map of LULC derived from ESA Sentinel-2 imagery of 2017 at 10 m resolution. The LULC data from ESRI showed 85% accuracy. The elevation of the study area has been extracted from Advanced Spaceborne Thermal Emission and Reflection Radiometer (ASTER) Global Digital Elevation Model (GDEM) datasets (https://asterweb.jpl.nasa.gov/gdem.asp, accessed on 25 January 2022).

### 2.3. Fish Sampling

Fish sampling was conducted from 2011 to 2019, before (April–May) and after (September–October) the monsoon period, when water levels were lower. Fish sampling was performed at 15 sites in the Geum River in accordance with the Ohio EPA (1989) [45] method, which was adapted for regional purposes by An et al. (2001) [46]. Fish populations were sampled overnight by using fyke nets (20 m long and 2.4 m high; mesh size, 5 × 5 mm), gill nets (50 m long and 2 m high; mesh size, 45 × 45 mm), and trammel nets (50 m long and 1.0 m high; mesh size, 12 × 12 mm). Cast nets (7 × 7 mm mesh size) and kick nets (4 × 4 mm mesh size) were utilized to catch fish in the run, riffle, and pool. A small boat was used to place the fyke nets, gill nets, and trammel nets along the river bank; cast and kick nets were placed at shallow sites in the river. The sampling stretch at each study site was 200 m, and the sampling duration was 60 min. After sample collection, the fish were identified and any abnormalities were documented. Previous regional research was used to determine the fish trophic and tolerance guilds [31].

### 2.4. Multi-Metric WPI

The multi-metric WPI was developed to assess the chemical health statuses of rivers and streams [8,47]. It consists of the following seven metrics that represent salient water quality factors: M_1_, TN (mg/L); M_2_, TP (μg/L); M_3_, TN:TP ratio; M_4_, BOD (mg/L); M_5_, TSS (mg/L); M_6_, EC (μS/cm); and M_7_, CHL-a (μg/L). Each metric in the WPI is awarded a score of 5, 3, or 1 depending on the concentration of the respective water quality factor (Tables S3 and S4; Supplementary file). The river’s overall chemical health is then determined by summing the scores of all metrics; the water quality status is classified as excellent (31–35), good (25–29), fair (19–23), poor (13–17), or very poor (7–11).

### 2.5. Multi-Metric IBI

The multi-metric IBI assesses a river’s biological health based on the fish community structure and fish abundance. It was regionally developed by An et al. (2006) [48] and consists of the following eight metrics: M_1_, total number of native fish species; M_2_, number of riffle benthic species; M_3_, number of sensitive species; M_4_, proportion of individuals that are tolerant species; M_5_, proportion of individuals that are omnivorous; M_6_, proportion of individuals that are native insectivorous species; M_7_, total number of native individuals; and M_8_, proportion of individuals with anomalies. Each metric is allocated a score of 5, 3, or 1 based on fish composition (Tables S5 and S6; Supplementary file). The overall biological health status of the river is obtained by summing the scores of all metrics; the health status is categorized as excellent (36–40), good (28–34), fair (20–26), poor (14–18), or very poor (8–13).

### 2.6. The QFCI Model

Stark (1985) first proposed the quantitative community index model to determine the health statuses of freshwater ecosystems, based on macroinvertebrate tolerance scores. Here, we adopted a version of the quantitative community index model based on fish tolerance scores [49] and renamed it the QFCI model. Based on QFCI scores, an ecosystem can be classified as excellent (0 to <2), good (2 to <4), fair (4 to <6), poor (6 to <8), or very poor (8 to 10). QFCI scores can be calculated by using the following formula:

$$\mathrm{QFCI}=\overset{i=S}{\underset{i=1}{\sum }}\frac{n_{i}\times a_{i}}{N}$$

where *S* is the total number of species in the sample, $n_{i}$ is the abundance of the ith scoring species, $a_{i}$ is the tolerance score for the ith species, and *N* is the total abundance in the entire sample.

### 2.7. Statistical Analyses

The LULC, elevation, water quality data, fish trophic and tolerance guilds, and IBI and QFCI scores were log_10_ converted to enhance data normality before the construction of empirical models. SigmaPlot software was used to perform linear regression analyses that could identify causal links among water chemistry and LULC, elevation, fish trophic and tolerance guilds, and IBI and QFCI scores. Box plots of analysis of variance and Tukey’s test results were used to visualize the spatial, seasonal, and annual variations of water quality variables in the Geum River using R version 4.1.1. The letters “a”, “b”, “c”, “d” and “e” in the box plots were derived from the *p*-values and were assigned in accordance with simple rules: the first highest mean receives the letter “a”, the second, third, fourth, and fifth highest mean receives the letter “b”, “c”, “d” and “e”, respectively, and means with no significant difference receive the same letter. The Mann–Kendall test was used to assess the temporal trends of water quality factors; this test was performed in ProUCL (ver. 5.1) software [50]. Canonical correspondence analysis (CCA) was used to examine the effects of LULC, water quality variables, and elevation on fish trophic and tolerance guilds in the river from 2011–2019. CCA was conducted by using PAST software [51].

## 3. Results

### 3.1. Site-Based, Seasonal, and Annual Variations in Water Quality

**Figure 2:**
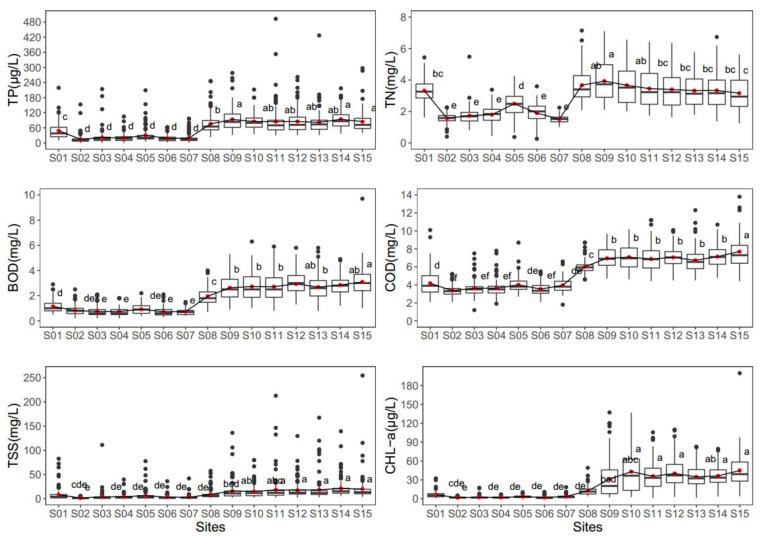
Site variations of water quality parameters in the Geum River basin (TP, total phosphorus; TN, total nitrogen; BOD, biological oxygen demand; COD, chemical oxygen demand; TSS, total suspended solids; CHL-a, chlorophyll-a. The red dots indicate the mean value, the first highest mean receives the letter “a”, the second, third, fourth, and fifth highest mean receives the letter “b”, “c”, “d” and “e”, respectively, and means with no significant difference receive the same letter).

Nutrients (TP and TN), organic matter (BOD and COD), TSS, and algal chlorophyll varied from site S01 to site S15 (Figure 2). The mean TP varied from 14.7 to 94.0 µg/L among sites S01–S15. Site S14 had the highest TP content (94 µg/L), whereas S02 (14.7 µg/L) had the lowest. TN, BOD, COD, TSS, and CHL-a were consistently lower at sites S02–S07 than at other sites. Dodds et al. (1998) [52] suggested that TP levels > 75 µg/L were indicative of eutrophic water in rivers. Mean TP levels > 75 µg/L were observed at sites S08–S15. However, the mean TN ranged from 1.51 to 3.93 mg/L among river sites, indicating N-rich systems. According to Dodds et al. (1998) [52], rivers with a mean TN content > 1.5 mg/L are considered eutrophic. High BOD and COD levels indicate the presence of organic pollution in aquatic systems. The mean BOD level in the Geum River varied from 0.72 to 3.09 mg/L, with the greatest concentration at site S15. Water with a COD level > 7 mg/L may only be utilized for industrial applications [53]; COD levels > 7 mg/L were detected at sites S10, S12, S14, and S15. The TSS content in the river originated from natural erosion and sediment transport [41]. Site S14 had the highest mean TSS concentration (21.2 mg/L). The CHL-a concentration is a critical factor that controls riverine ecosystem eutrophication. The mean CHL-a concentration varied from 1.81 to 44.9 µg/L in the river. The highest CHL-a concentration was detected at site S15 (44.9 µg/L). Dodds et al. (1998) regarded CHL-a concentrations > 30 µg/L as eutrophic. The mean CHL-a concentration was >30 µg/L at sites S09, S10, S11, S12, S13, S14, and S15.

There were substantial seasonal fluctuations in WT, DO, BOD, COD, TP, TN, TSS, EC, and sestonic CHL-a (Figure S2; Supplementary file). Summer had the greatest mean WT (24.3 °C) among the four seasons. Because of seasonal influences, DO levels in the river were generally low (9.13 mg/L) in summer and high (13.5 mg/L) in winter. The diluting effect of the monsoon caused summer to have the lowest TN (2.09 mg/L) and EC (207 S/cm). The mean BOD was highest in spring (2.30 mg/L) because of the low river flow. The mean COD (6.09 mg/L), TSS (20.1 mg/L), and TP (76.2 µg/L) levels were higher in summer than in other seasons because of the increased flow of water in the river. The CHL-a concentration was also higher in summer (24.6 µg/L).

Annual changes can provide insights into the long-term dynamics of water quality in aquatic systems (Figure S3; Supplementary file). The highest TP (90.3 µg/L) and TN (3.13 mg/L) levels were observed in 2011. The organic matter content in the water body increased over time; the highest levels were observed in 2019 (BOD: 2.21 mg/L and COD: 5.90 mg/L). The CHL-a concentrations were also highest in 2016 and 2019 (25.7 and 24.1 µg/L). Additionally, the Mann–Kendall test was used to assess long-term changes in water quality parameters from 2011 to 2019. These findings are reported in Table S1 (Supplementary file). The organic matter (BOD and COD), algal chlorophyll (CHL-a), and ionic level (EC) in the river displayed an increasing trend; other parameters did not exhibit any trend.

### 3.2. Relationships between LULC and Water Chemistry

**Figure 3:**
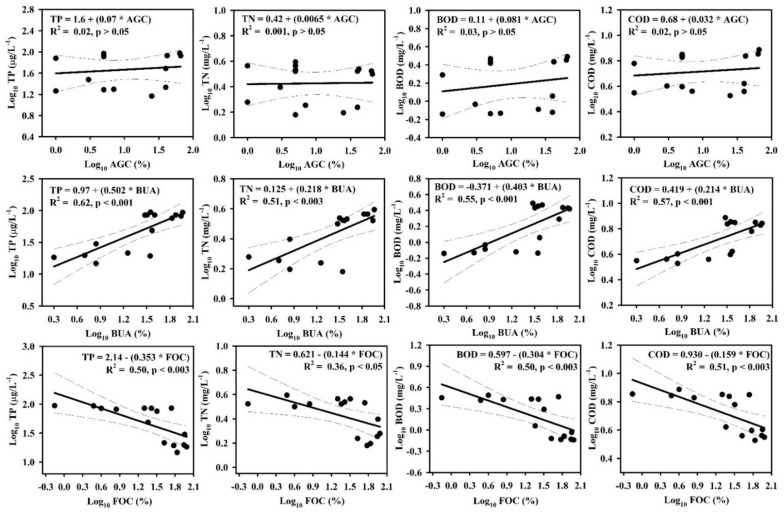
Relationships of LULC with water quality variables (AGC, agricultural coverage; BUA, built-up area coverage; FOC, forest coverage; TP, total phosphorus; TN, total nitrogen; BOD, biological oxygen demand; COD, chemical oxygen demand).

Regression analysis was conducted to determine the relationships of LULC with nutrients and organic matter. The results revealed distinct effects of LULC on TP, TN, BOD, and COD in the river basin (Figure 3). Agricultural and built-up area cover directly influenced TP, TN, BOD, and COD, while forest cover had a negative effect. Agricultural cover was not significantly (*p* > 0.05) correlated with TP, TN, BOD, or COD levels in the river, but the built-up area was strongly correlated with TP (R^2^ = 0.62, *p* < 0.001), TN (R^2^ = 0.51, *p* < 0.003), BOD (R^2^ = 0.55, *p* < 0.001), and COD (R^2^ = 0.57, *p* < 0.001) levels. The TP (R^2^ = 0.50, *p* < 0.003), TN (R^2^ = 0.36, *p* < 0.05), BOD (R^2^ = 0.50, *p* < 0.003), and COD (R^2^ = 0.51, *p* < 0.003) levels were substantially reduced as the forest cover increased. Elevation also influenced the TP, TN, BOD, COD, TSS, and CHL-a levels. As elevation increased, the TP (R^2^ = 0.63, *p* < 0.001), TN (R^2^ = 0.39, *p* < 0.05), BOD (R^2^ = 0.77, *p* < 0.001), COD (R^2^ = 0.84, *p* < 0.001), TSS (R^2^ = 0.66, *p* < 0.001), and CHL-a (R^2^ = 0.80, *p* < 0.001) levels decreased (Figure S4; Supplementary file).

### 3.3. Suspended Solids, Nutrients, and Chlorophyll-A Dynamics

**Figure 4:**
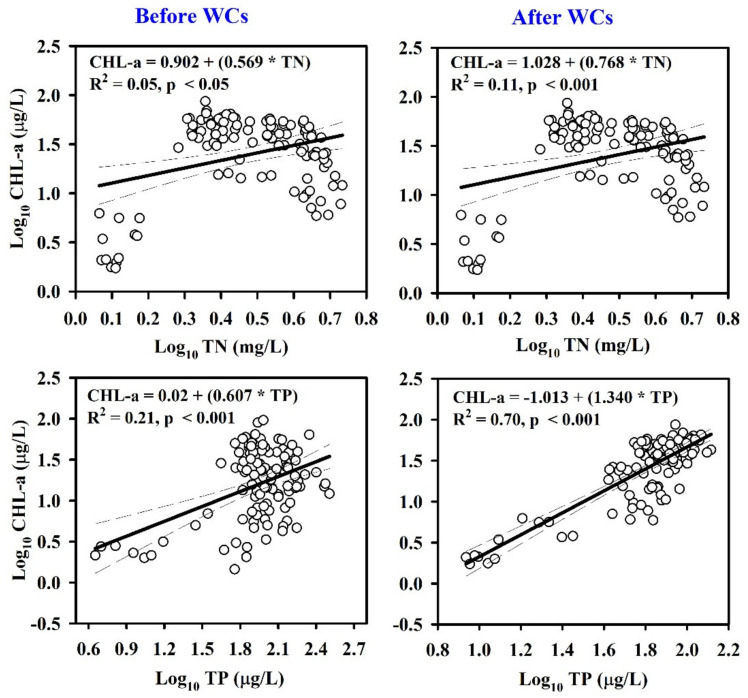
Relations of algal chlorophyll (CHL-a) with total nitrogen (TN) and total phosphorus (TP) in the Geum River before weir construction (WCs) and after the weir construction.

The interactions between SS and nutrients were investigated via regression analysis (Figure S5; Supplementary file). The results indicated that TSS was a stronger predictor of TP (R^2^ = 0.83) than TN (R^2^ = 0.13) in the Geum River. Furthermore, the algal CHL-a concentration was significantly controlled by TP and TN; it was seasonally dependent on the Geum River (Figure S6; Supplementary file). In winter, spring, summer, and fall, TP was a greater regulator of CHL-a than TN. The entire river empirical model also suggested that TP (R^2^ = 0.78) was a better predictor of CHL-a than TN (R^2^ = 0.31). Additionally, empirical models were developed that link nutrients and algal CHL-a at two time points (i.e., before and after weir construction) to determine the effect of weirs on algal growth (Figure 4). Before weir installation, TP had a negligible influence on algal CHL-a in the river (R^2^ = 0.21). In contrast, the construction of the weir transformed the river system into a series of lakes, indicating that TP (R^2^ = 0.70) was the primary regulator of algal production in the river. The TN:TP ratio is widely regarded as an indicator of nutrient limitation effects on algal CHL-a. According to empirical data, a large TN:TP ratio suggests a greater likelihood of P-limitation (Figure S7; Supplementary file). An increase in the TN:TP ratio significantly reduced the CHL-a concentration (R^2^ = 0.68), indicating P-limitation.

### 3.4. Fish Fauna and Guild Composition

The assessment of fish species composition at all river sites (S01–S15) revealed substantial changes in relative abundance (RA), total number of individuals, total number of species, and fish guilds (tolerance, trophic, and habitat), as shown in Table S2 (Supplementary file). The most commonly sampled fish was *Zacco platypus*, which was the dominant river fish species with 5696 individuals and a RA of 25.62%. Based on RA, the most abundant fish species (in decreasing order) were *Z. platypus*, *Pseudogobio esocinus*, *Zacco koreanus*, *Pungtungia herzi*, *Hemibarbus labeo*, and *Anacampseros lanceolota*, which together comprised 51.14% of all fish collected. In the river, four endangered (*Pseudopungtungia nigra*, *Gobiobotia brevibarba*, *Gobiobotia macrocephala*, and *Gobiobotia nakdongensis*) and three exotic (*Micropterus salmoides*, *Lepomis macrochirus*, and *Carassius cuvieri*) fish species were observed during the study period. The numbers of sensitive and riffle benthic species significantly varied from site S01 to site S15. Sensitive species were absent from sites S12, S14, and S15. The RA of riffle benthic (R^2^ = 0.40) and sensitive species (R^2^ = 0.82) increased with increasing elevation (Figure S8; Supplementary file). Analysis of the annual variations of fish guilds indicated that the RAs of stagnant (R^2^ = 0.28), exotic (R^2^ = 0.04), tolerant (R^2^ = 0.37), and carnivorous (R^2^ = 0.24) species increased, but the RA of sensitive species (R^2^ = 0.44) decreased during the study period (Figure S9; Supplementary file).

### 3.5. Spatial and Temporal Assessments Based on the WPI, IBI, and QFCI Models

**Figure 5:**
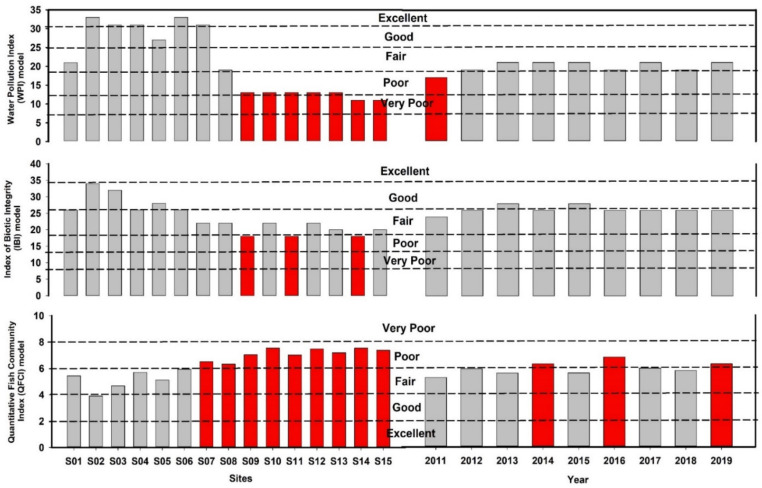
Chemical and biological health status of the Geum River basin based on sites and year (red bars indicate “poor” and “very poor” condition).

The WPI, IBI, and QFCI scores displayed varying degrees of alteration depending on the study site and year (Figure 5). Overall, WPI scores ranged from 11 to 33, indicating that the river’s chemical health was poor to excellent (Table S3; Supplementary file). The WPI results indicated that the chemical health statuses of two sites (S14 and S15) were “very poor”, while the statuses of other sites were “poor” (five sites: S09, S10, S11, S12, and S13), “fair” (two sites: S01 and S08), “good” (one site: S05), or “excellent” (five sites: S02, S03, S04, S06, and S07). The annual WPI results indicated that the river chemical health was in a fair to poor condition from 2011 to 2019 (Table S4; Supplementary file). The IBI score varied from 18 to 34, indicating that the river’s biological health was poor to good (Table S5; Supplementary file). The IBI results suggested that biological health was good at three sites (S02, S03, and S05); at other sites, it was fair (nine sites: S01, S04, S06, S07, S08, S10, S12, S13, and S15) or poor (three sites: S09, S11, and S14). The annual IBI results indicated that the river’s biological health was fair to poor (Table S6; Supplementary file). In contrast, the biological health assessment based on the QFCI suggested that only one site (S02) was in a “good” condition, and the conditions of other sites were “fair” (five sites: S01, S03, S04, S05, and S06) or “poor” (nine sites: S07–S15) (Figure 5). The annual QFCI results indicated a fair to poor biological health status. The WPI score was significantly correlated with the IBI (R^2^ = 0.66) and QFCI (R^2^ = 0.70) scores of the river (Figure S10; Supplementary file). The IBI and QFCI scores were strongly correlated with each other (R^2^ = 0.87).

### 3.6. Relationships among Water Chemistry, LULC, Trophic and Tolerance Guilds, and Models

**Figure 6:**
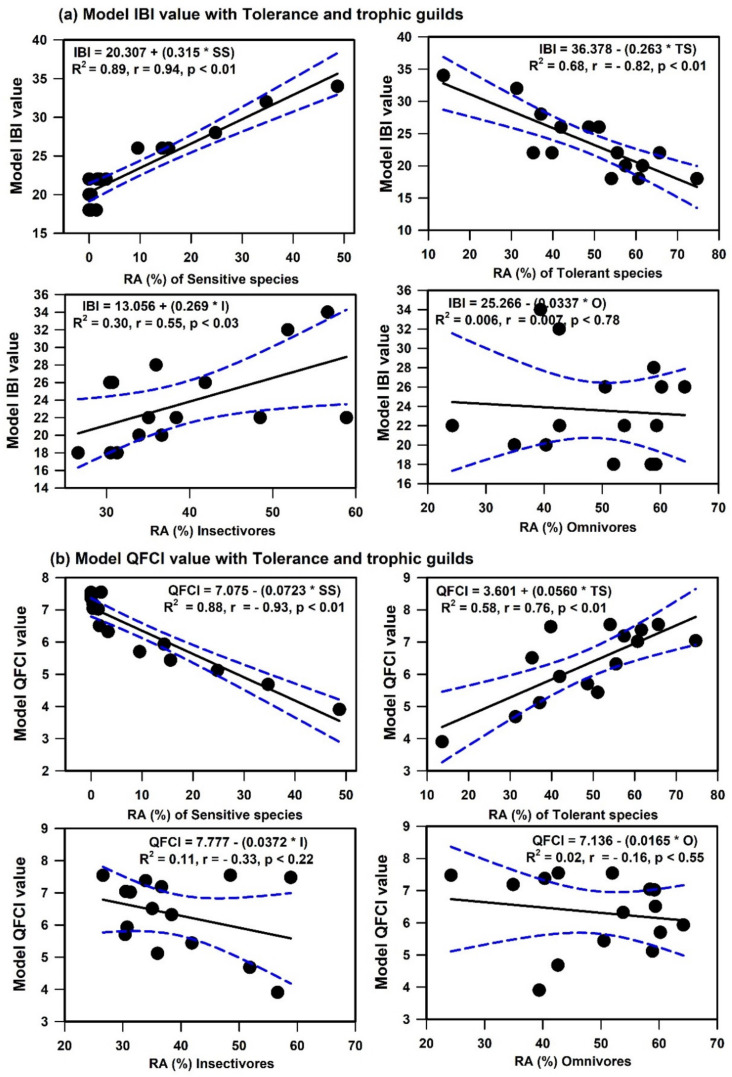
Relations among the Index of Biotic Integrity (IBI) and Quantitative Fish Community Index (QFCI) model values with tolerant and trophic guild.

The river nutrients (TP and TN), organic matter (BOD and COD), and algal CHL-a all affected the RAs of trophic and tolerance guilds (Table 1). The RAs of carnivorous and tolerant species were positively correlated with TP, TN, BOD, COD, and CHL-a; however, those RAs were negatively correlated with the RAs of omnivorous, insectivorous, sensitive, and intermediate species. Regression analyses revealed that nutrient enrichment, organic pollution, and algal blooms significantly influenced the RAs of carnivorous (TP: R^2^ = 0.57, TN: R^2^ = 0.38, BOD: R^2^ = 0.64, COD: R^2^ = 0.65, CHL-a: R^2^ = 0.65) and tolerant (TP: R^2^ = 0.55, TN: R^2^ = 0.57, BOD: R^2^ = 0.41, COD: R^2^ = 0.49, CHL-a: R^2^ = 0.47) fish species. In contrast, the RA of sensitive species declined dramatically when the river TP (R^2^ = 0.62), TN (R^2^ = 0.42), BOD (R^2^ = 0.66), COD (R^2^ = 0.74), and CHL-a (R^2^ = 0.74) levels increased. Additionally, nutrients, organic pollutants, and algal blooms substantially impacted the biological health of Geum River. The IBI score decreased with increasing TP (R^2^ = 0.66), TN (R^2^ = 0.50), BOD (R^2^ = 0.65), COD (R^2^ = 0.73), and CHL-a (R^2^ = 0.71) levels. The QFCI score declined as nutrients, organic matter, and primary productivity increased, indicating worsening biological health conditions. The IBI score significantly increased as the RAs of sensitive and insectivorous species increased, but it decreased as the RA of tolerant species increased (Figure 6). The QFCI score decreased as the RAs of sensitive and insectivorous species increased, but it increased as the RA of tolerant species increased.

The LULC was also a strong determinant of fish guilds and biological health (Table 1). Regression analysis revealed that the built-up area was substantially correlated with increased RAs of carnivorous (R^2^ = 0.29) and tolerant (R^2^ = 0.30) fish species. The RA of sensitive fish (R^2^ = 0.55) decreased as the built-up area increased. The built-up area had a detrimental effect on the river’s biological health (IBI: R^2^ = 0.42, QFCI: R^2^ = 0.37). The ecological integrity (IBI: R^2^ = 0.49, QFCI: R^2^ = 0.33) and RA of sensitive fish (R^2^ = 0.59) were enhanced with increasing forest cover, whereas the RAs of tolerant (R^2^ = 0.29) and carnivorous (R^2^ = 0.53) fish declined with decreasing forest cover. However, agricultural land cover did not significantly influence fish guilds or river ecological health.

### 3.7. Insights from CCA

**Figure 7:**
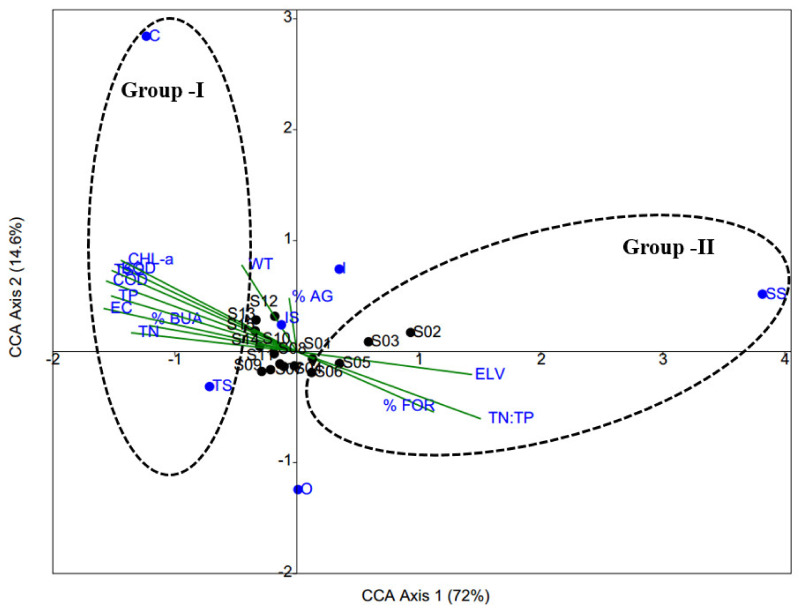
Canonical correspondence analysis (CCA) ordination diagram of trophic and tolerance guilds with water quality variables and land-use pattern and elevation. IS, intermediate species; SS, sensitive species; TS, tolerant species; C, carnivore; I, Insectivores; O, omnivores; AG, agricultural land-use coverage; FOR, forest land-use coverage; BUA, built-up area; WT, water temperature; BOD, biological oxygen demand; COD, chemical oxygen demand; TSS, total suspended solids; TP, total phosphorus; TN, total nitrogen; CHL-a, chlorophyll-a; EC, electrical conductivity.

Although CCA results could explain 86.6% of the total variance on the first two axes, permutation testing indicated that only axis 1 (*p* < 0.04) was statistically significant (Figure 7 and Table S7; Supplementary file). CCA was used to ascertain the effects of environmental variables on trophic and tolerance guilds in the river fish community. We discovered two distinct groupings of variables (Figure 7). The first group was defined by highly significant, environmentally relevant variables: TP, TN, BOD, COD, TSS, EC, CHL-a, and built-up area (%). These variables were located on the left side of the CCA graph and were correlated with the RAs of carnivorous and tolerant fish species. The second group included forest cover (%), elevation, and the TN:TP; these variables were correlated with the presence of sensitive species. Thus, the presence of carnivorous and tolerant species within the fish community was suggestive of worsening ecological health; the presence of sensitive species in the river was indicative of greater ecological integrity.

## 4. Discussion

### 4.1. Zonal and Temporal Water Quality Variations

The water quality of the river channel significantly varied between headwater and downstream sites. The headwater sites (S01–S07) had better water quality, whereas factors associated with impaired water quality status were increased at downstream sites (S08–S15). The upper portions of the river were densely forested, indicating good water quality. In contrast, the downstream section was surrounded by towns, cities, and agricultural land, which directly contributed to water quality degradation. Moreover, weir construction regulated the riverine transit of suspended particles, nutrients, and organic matter [40]. Eutrophication was accelerated in downriver sites (S08–S15) due to enhanced inflows of TP and TN. Increased BOD and COD levels lead to the degradation of water quality. A body of water with a high TSS is presumably contaminated, either naturally or via human activities. There was an increase in the sestonic CHL-a concentration, particularly in downriver sites (S09–S15), after weir construction. Algal CHL-a-based eutrophic river conditions can decrease the DO concentration and negatively impact inherent ecological processes. Our findings indicate that immediate action is required to minimize anthropogenic pollution in the main channel of the Geum River, especially at sites S08 to S15.

More than 60% of the annual precipitation in Asia occurs in summer, which is the monsoon season [2,8]. This weather cycle triggers typical water quality patterns at seasonal and annual scales that are inextricably linked to the longitudinal morphology of the river ecosystem. Summer monsoon rainfall boosts the flow regime (inflow and outflow) in the Asian watersheds. It reduces WRT and regulates nutrients, organic matter, ionic levels, suspended particle loadings, and algal growth. It can also significantly impact the functional links among water quality factors in an ecosystem. Our analysis demonstrated that the TP, TSS, COD, and CHL-a levels were higher in summer than in other seasons, suggesting that the summer monsoon may affect water quality. Additionally, the elevated TSS level in summer was attributed to an increased TP concentration in the river. The TP, TSS, and COD inputs are related to the flow regime of the Asian river basin [3,5,11]. Thus, increased TP input during the monsoon season may affect river algal growth. Summer had the greatest mean WT value. Because of the seasonal effect, a strong negative relationship was observed between WT and DO. There was a natural inverse relationship because warmer water absorbs oxygen more rapidly and stores less DO. The diluting effect of the summer monsoon decreased the mean EC and TN values in the river [2,8].

An analysis of the annual variations of water quality parameters can offer valuable insights into long-term water quality dynamics in aquatic ecosystems. Here, we observed increasing organic matter and algal CHL-a concentrations in the river. Our findings indicated that structural modifications in the physical environment had resulted in altered water chemistry. Mann–Kendall tests revealed that BOD, COD, CHL-a, and EC levels increased, but other parameters (WT, TSS, TN, and TP) remained stable. Despite steady inputs of TP and TN, the river remained in a eutrophic state, indicating that Asian temperate rivers are nutrient-rich systems. The BOD and COD levels in the river displayed a declining trend, suggesting that the organic matter content in the river increased due to substantial inflow of various pollutants (point source and non-point source) [2]. Increasing retention of organic matter in impounded rivers has been reported in many freshwater ecosystems worldwide [54]. The CHL-a concentration and EC in the river were increased because of enhanced WRT and decreased outflow. Increased algal biomass and ion concentrations have also been documented from numerous freshwater ecosystems that have been impounded by dams or weirs [55,56]. These organic, ionic, and algal pollutants can cause various water quality problems, which impact drinking, domestic, agricultural, and industrial water deliveries.

### 4.2. The Impacts of LULC and Elevation on Water Quality

Human activities are the primary cause of LULC modifications globally; they influence lotic systems and associated watersheds [57,58,59]. Land use and land cover is a critical landscape component that changes the hydrological and physicochemical characteristics of rivers [60]. Researchers have acknowledged that rivers and streams are primarily influenced by the LULC of the surrounding landscape [59,61]. The land use patterns of a river basin regulate the transportation of nutrients, organic matter, pollutants, and the sediments of recipient waters by modifying the flows of surface water and groundwater, inputs of organic matter, and atmospheric deposition into the river [57,60]. Our analysis showed that river water quality is firmly linked to LULC. In the forest area, river water had low nutrient and organic matter concentrations; these concentrations were higher in agricultural and built-up areas. Many other studies have reported a tendency for river water chemistry to be better in forested regions than in regions with other types of land use; higher nutrient and organic matter concentrations have been reported in agricultural and built-up areas [2,5,39,58]. Allan et al. (1997) [59] reported that the increased TN, TP, BOD, and COD levels in river water were the immediate consequence of increases in agricultural and built-up areas, whereas reductions in these levels were related to an increase in forest cover. The percentage of agrarian LULC at the catchment scale is a significant predictor of TN, TP, BOD, and COD. However, in the current study, the percentage of built-up area at the catchment scale was the primary predictor of nutrient and organic matter concentrations. The urban landscape is a complex assortment of areas that support various human activities, including industry, transportation, housing, commerce, government infrastructure, communication and utilities, and recreation. Previous studies have shown that an increase in the built-up area will lead to greater impervious surface coverage [62]. The water quality deterioration through built-up areas is correlated with the levels of point source pollutants from industrial and business operations, combined with high levels of domestic sewage [63]. Diffused urban surface run-off is also responsible for river water pollution. Nutrients and oxygen-demanding organic materials are frequently built from diffused urban sources during the dry months; they are transported in large quantities to river channels during concentrated rainfall events [63].

Elevation was also an excellent indicator of water quality in the river. Our study showed that nutrients, organic matter, SS, and algal chlorophyll increased with decreasing elevation. These findings were consistent with the assumption that low elevation reaches have higher levels of nutrients, organic matter, and solids, with a greater algal biomass than higher elevations [2,64]. Furthermore, elevation indirectly defines the intensity and nature of land use; it is linked to the release of pollutants [64]. Industrialization, urbanization, and farming were mainly observed in areas of flat topography, whereas forests were dominant in higher elevation zones [65].

### 4.3. Solids, Nutrients, and Algal Chlorophyll Dynamics

Nutrients (TP and TN) can enter river systems through point and non-point sources. Most TP and TN inputs are linked to sediments during run-off [66]. According to Sharpley et al. (1993), 75–90% of TP might be bound to SS. The significant linkage among TP, TN, and TSS has a detrimental effect on water quality [67]. This pollutant-transport role implies that TSS can act as a carrier of TP and TN. The current findings indicate that TSS might operate as a significant carrier of TP in the river basin, but not as a carrier of TN. According to Hutchinson (1957) [68], “phosphorus is the most critical element for the ecologist because it is more likely to be deficient, thus limiting the biological productivity of any region of the earth’s surface more than the other major biological elements.” This assertion prompted the development of empirical models involving trophic state parameters that could be used in eutrophication management [32,33,69]. An earlier study of algal biomass and nutrients emphasized lentic ecosystems and indicated that TP was the key predictor of algal development [69]. This type of prediction has also been applied to lotic systems; however, TP is a poor indicator of algal growth in lotic waters because of seasonality and the complex interactions of water quality with environmental factors (e.g., nutrients, organic matter, WT, light availability, flow, habitat conditions, invertebrate, and fish feeding grounds) [70,71,72]. The link between TP and algal biomass in lotic systems displays substantial fluctuation; therefore, the relationship is inappropriate for eutrophication management. However, the current findings are consistent with reports by Hutchinson (1957) and Dillion (1974) that the river has changed from a lotic to a lentic system since the installation of three massive weirs. Weir construction has increased the WRT and reduced outflow from the water column, suggesting that TP is the primary factor that governs algae growth. These findings corroborate previous findings in river systems that are regulated by weirs [55,56].

Additionally, the TN:TP ratio has been used to determine the status of algal nutrient limitation. P-limitation has been considered more likely in systems with a higher TN:TP ratio [32]. The present study indicated a tendency for CHL-a concentrations to decrease as the TN:TP ratio increased, implying a significant P-limitation. After a review of 157 published papers, Yan et al. (2016) [73] concluded that the TN:TP ratio is negatively associated with CHL-a worldwide.

### 4.4. Water Quality and LULC Impacts on Fish Guilds and Biological Health

The multi-metric indices of WPI, IBI, and QFCI indicated distinct longitudinal variations in chemical and biological integrity from headwater sites to downstream sites. The WPI model suggested that the chemical health of the downstream area was “poor” or “very poor”. The biological health assessment also reflected these zonal differences in chemical health. The biological health of the downstream area was “fair” or “poor”, with a skewed trophic structure and skewed distributions of carnivores and omnivores; tolerant species were common and sensitive species were rare. This result is consistent with the findings of some previous regional investigations concerning biological integrity [2,5,8,14]. In addition, the annual WPI scores matched the annual IBI and QFCI scores.

Fish trophic and tolerance guilds can be used to estimate the impacts of water quality on the riverine food chain and ecological integrity. The present study revealed that nutrient enrichment, organic pollution, and algal blooms significantly enhanced the mean RA of carnivores. A similar relationship was observed for the tolerant species in this lotic ecosystem. Consequently, the RA of sensitive species declined with increasing TP, TN, BOD, COD, and CHL-a. These findings support the hypothesis that increases in tolerant and carnivorous species will damage river ecological quality and contribute to the extinction of sensitive species [4,14,31]. Overall, the relationships between biological health and chemical parameters, as well as the trophic and tolerance compositions, suggested that river health is closely related to nutrient enrichment, organic pollution, and algal growth; moreover, chemical conditions dictate the trophic and tolerance compositions of Asian lotic ecosystems.

The relationship between LULC and both fish community structure and biological health is complex on a catchment scale. According to Allan (2004) [58], a decline in forest cover and increases in agricultural and urban land cover will have negative effects on river habitats and biota. Roth et al. (1996) [74] reported that agricultural land was the critical determinant of the prevailing structures of fish communities in rivers; it was negatively associated with IBI outcomes. Consequently, a damaged fish community was a direct indication of increasing agricultural land use. However, Wang et al. (1997) [75] did not identify any functional relationship between IBI and agricultural land use patterns, with the exception of study regions where agricultural land use was the primary land use pattern within the river basin. These authors proposed that fish populations may not respond to lower agricultural coverage within a basin. The results of the present study concurred with findings by Wang et al. (1997) [75]; thus, a built-up area with an impervious surface may be the most significant predictor of fish guilds and may substantially influence biological health. Numerous studies in urban rivers have shown that river conditions respond nonlinearly to impervious surfaces, and severe degradation occurs in 15–25% of rivers in areas with urban land cover [76]. In the present study, ecological integrity increased with increasing forest cover. Numerous studies have shown that biological health improves when forest cover increases in the basin [1,2,31,58,63]. CCA results also suggested that chemical pollution and the built-up area were responsible for the higher RAs of carnivores and tolerant species, indicating poor ecological integrity in the downstream area of the river. It also implied that headwater regions were less affected by chemical degradation because of forest cover; such regions could support a greater number of sensitive species, indicating robust ecological integrity.

### 4.5. Link between Ecological and Human Health

There is a stronger connection between ecological and human health. Nutrients, WRT, and temperature impacted algal growth and toxin production in the aquatic systems [77]. Previous research documented that microcystis and microcystins levels are favorably linked with the CHL-a level in most Korean rivers [78,79]. Lee et al. [77] reported a significant association between harmful algal blooms (HABs) intensity and the incidence of nonalcoholic liver diseases in Korea. Several researchers acknowledged a statistically significant relationship between HABs intensity and liver disease or cancer in China, the United States (US), and Serbia [80,81,82]. In the United States, liver disease mortality amplified by 0.3% for every 1% upsurge in bloom coverage in the affected county [80]. Moreover, poor chemical integrity destroys crops and contaminates food, posing a hazard to aquatic and human life as well as interrupting the food chain and causing respiratory problems in fish [83,84]. Pollution-contaminated gills are fatal to fish. Consuming this fish causes serious health issues, including respiratory problems, cancer, diarrhea, neurological disorders, and cardiovascular disease [80,81,83]. Due to an excessive accumulation of nutrients, organic matter, and algal and ionic content, the current investigation found poor chemical and biological health in the river’s downstream region. Consistent with the previous study, our data demonstrate that pollutants significantly impact downstream water [84,85,86].

## 5. Conclusions

The present study evaluated ecological river health based on multi-metric indices of WPI, IBI and QFCI. The LULC and elevation determined the nutrients, organic matter, ions, and SS in the river systems. A high percentage of built-up area increased the watershed nutrient and organic matter inputs. In contrast, an increasing forest cover rate resulted in lower TN, TP, BOD, and COD levels in the river ecosystem. Moreover, the pollutant transport theory suggested that TSS is a good predictor of TP loading in the river. The nutrient regimes were directly linked to algal biomass and trophic interactions. The empirical models suggested that TP was the critical factor regulating algal growth. The TN:TP ratios indicated P-limitation and an N-rich system. Longitudinal gradient analyses of nutrients, organic matter, suspended solids, and algal CHL-a revealed severe chemical pollution in the downstream area because of run-off from point and non-point sources. These zonal differences in chemical health were directly linked to biological health. Annual variations and Mann–Kendall test results suggested that levels of organic matter and algal CHL-a tended to increase because of weir construction.

Nutrients, organic matter, and primary productivity also determined the fish guilds (trophic and tolerance) and biological health. The trophic relationships among water quality factors and fish guilds suggested that the RAs of tolerant and carnivorous fish species were significantly positively correlated with nutrients, organic matter, and algal CHL-a. In contrast, the RA of sensitive fish species was significantly negatively correlated with nutrients, organic matter, and algal CHL-a. Our findings indicated that nutrient and organic matter concentrations directly shaped fish guilds (trophic and tolerance) and primary productivity (CHL-a); these concentrations were closely associated with LULC, point source, and elevation. Notably, these physicochemical parameters regulated the river’s ecological health. A river health assessment based on fish assemblages indicated that the IBI and QFCI scores were significantly affected by watershed land use features (LULC), nutrient levels (TN and TP), organic matter indicators (BOD and COD), primary productivity (CHL-a), and biological components (trophic and tolerance guilds). Overall, the river health evaluation indicated that the downstream area of the river was in poor ecological condition; it requires immediate and comprehensive restoration measures.

## Figures and Tables

**Table 1:** Coefficients of determination (R^2^) for regressions among LULC, nutrients, organic matter, algal chlorophyll with trophic and tolerance guilds and model IBI and QFCI value in the Geum River (* indicates significant at *p* < 0.05; (+) indicates the relationship is positive; and (−) indicates it is negative; TN, total nitrogen; TP, total phosphorus; BOD, biological oxygen demand; COD, chemical oxygen demand; CHL-a, chlorophyll-a; LULC, land-use land-cover; % AG, percentage of agricultural coverage; % BUA, percentage of built-up area; % FOR, percentage of forest coverage).

Environmental Variables		Trophic Guilds			Tolerance Guilds			Model IBI Value	Model QFCI Value
		% Omnivores	% Insectivores	% Carnivores	% Sensitive species	% Intermediate Species	% Tolerant Species		
Nutrients	TN	0.04 (−)	0.02 (−)	0.38 * (+)	0.42 * (−)	0.10 (−)	0.57 * (+)	0.50 * (−)	0.47 * (+)
	TP	0.10 (−)	0.02 (−)	0.57 * (+)	0.62 * (−)	0.03 (−)	0.55 * (+)	0.66 * (−)	0.63 * (+)
Organic matters	BOD	0.17 (−)	0.01 (−)	0.64 * (+)	0.66 * (−)	0.01 (−)	0.41 * (+)	0.65 * (−)	0.63 * (+)
	COD	0.12 (−)	0.02 (−)	0.65 * (+)	0.74 * (−)	0.007 (−)	0.49 * (+)	0.73 * (−)	0.73 * (+)
Primary productivity	CHL-a	0.15 (−)	0.01 (−)	0.65 * (+)	0.74 * (−)	0.007 (−)	0.47 * (+)	0.71 * (−)	0.73 * (+)
LULC	% AG	0.03 (−)	0.001 (+)	0.06 (+)	0.05 (+)	0.03 (−)	0.007 (−)	0.002 (−)	0.006 (−)
	% BUA	0.10 (−)	0.007 (+)	0.29 * (+)	0.55 * (−)	0.02 (−)	0.30 * (+)	0.42 * (−)	0.37 * (+)
	% FOR	0.006 (+)	0.17 (+)	0.53 * (−)	0.59 * (+)	0.07 (+)	0.29 * (−)	0.49 * (+)	0.33 * (−)

## Data Availability

The data may be available upon request to the corresponding author, however, it is with subject to approval from the funding agency.

## Supplementary Materials

The following supporting information can be downloaded at: https://www.mdpi.com/article/10.3390/ijerph19159305/s1. Figure S1, Map showing the stream order of the Geum River basin; Figure S2, Seasonal variations of water quality parameters in the Geum River basin (WT: water temperature; DO, dissolved oxygen; BOD, biological oxygen demand; COD, chemical oxygen demand; TP, total phosphorus; TN, total nitrogen; TSS, total suspended solids; EC, electrical conductivity; CHL-a, chlorophyll-a; Spring, Mar-May; Summer, Jun-Aug; Fall, Sep-Nov; Winter, Dec-Feb, the red dots indicate the mean value); Figure S3, Yearly variations of water quality variables in the Geum River basin (TP, total phosphorus, TN, total nitrogen, BOD, biological oxygen demand; COD, chemical oxygen demand; TSS, total suspended solids; CHL-a, chlorophyll-a, the red dots indicate the mean value); Figure S4, Relations of nutrients (TP, total phosphorus; TN, total nitrogen), organic matters (BOD, biological oxygen demand; COD, chemical oxygen demand), total suspended solids (TSS), and chlorophyll-a with elevation (EV) in the Geum River basin; Figure S5, Relations of suspended solids with nutrients (TP, total phosphorus; TN, total nitrogen); Figure S6, Relations of algal chlorophyll (CHL-a) with total nitrogen (TN) and total phosphorus (TP) in the Geum River (Spring, March–May; Summer, June–August; Fall, September–November; Winter, December–February); Figure S7, Nutrient limitation status determination based on the empirical relationship of algal chlorophyll (CHL-a) with TN:TP ratios; Figure S8, Relations of riffle benthic and sensitive fish species with elevation; Figure S9, Yearly variations of stagnant, exotic, sensitive, tolerant, and carnivore fish species in the Geum River (the black line indicates the regression line); Figure S10, Relations among Water Pollution Index (WPI), Index of Biotic Integrity (IBI) and Quantitative Fish Community Index (QFCI) model values; Table S1, Mann–Kendall trend analysis of water quality parameters in the Geum River basin. (WT, water temperature; EC, electrical conductivity; TSS, total suspended solids; BOD, biological oxygen demand; COD, chemical oxygen demand; TP, total phosphorus; TN, total nitrogen; CHL-a, chlorophyll-a); Table S2, Fish fauna and guild composition in the Geum River watershed. (Tol.G., tolerance guild, Tro. G., trophic guild; Hab. G., habitat guild; RA, relative abundance; TNI, total number of individuals; TRA, total relative abundance; TNS, total number of species, ¥, exotic species, * endangered species; SS, sensitive species; IS, intermediate species; TS, tolerant species; O, omnivores; I, insectivores; C, carnivores; H, herbivores RB, riffle benthic species); Table S3, Site-based chemical health assessment (CHA) based on multi-metric water pollution index (WPI) in the Geum River basin. (EX, excellent; G, good; F, fair; P, poor; VP, very poor; Table S4, Yearly chemical health assessment (CHA) based on multi-metric water pollution index (WPI) in the Geum River basin. (F, fair; P, poor); Table S5, Site-based biological health assessment (BHA), based on the index of biotic integrity (IBIKR) using fish assemblages in the Geum River Basin. (F, fair; G, good; P, poor); Table S6, Yearly biological health assessment (BHA), based on the index of biotic integrity (IBIKR) using fish assemblages in the Geum River Basin (F, fair, G, Good); Table S7, Canonical correspondence analysis of trophic and tolerance guilds with water quality variables and land-use pattern and elevation; IS, intermediate species; SS, sensitive species; TS, tolerant species; C, carnivore; I, Insectivores; O, omnivores; AG, agricultural land-use coverage; WT, water temperature; BOD, biological oxygen demand; COD, chemical oxygen demand; TSS, total suspended solids; TP, total phosphorus; TN, total nitrogen; CHL-a, chlorophyll-a; EC, electrical conductivity (* *p* < 0.05).
