# Supplementary material for: Ecological River Health Assessment Using Multi-Metric Models in an Asian Temperate Region with Land Use/Land Cover as the Primary Factor Regulating Nutrients, Organic Matter, and Fish Composition
Source: Int J Environ Res Public Health. 2022 Jul 29;19(15):9305. doi: 10.3390/ijerph19159305 (PMC9368116; doi:10.3390/ijerph19159305)
Supplement: Supplementary file 1 [file ijerph-19-09305-s001.zip › ijerph-1792027-supplementary.pdf]

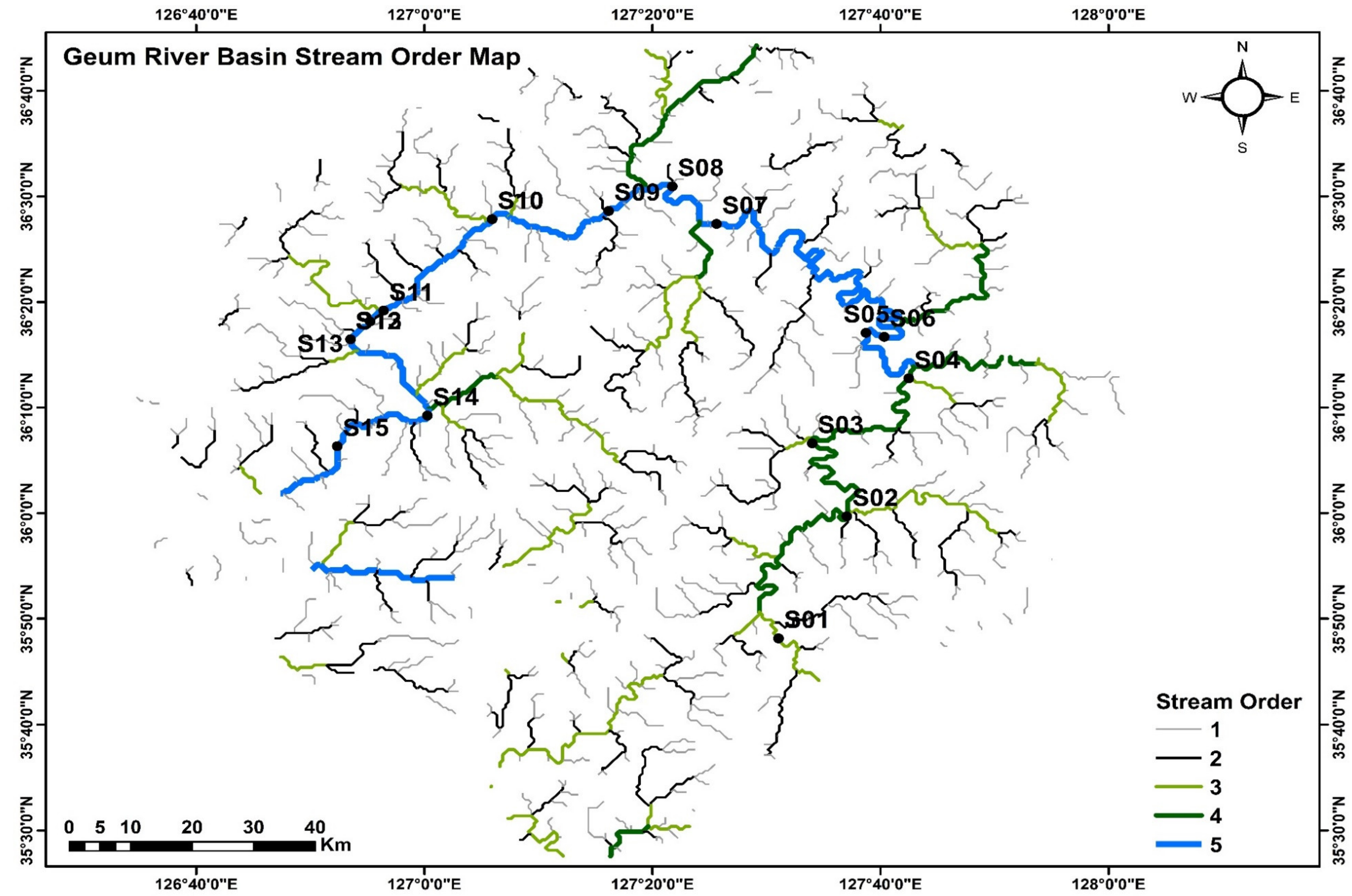

Figure S1. Map showing the stream order of the Geum River basin

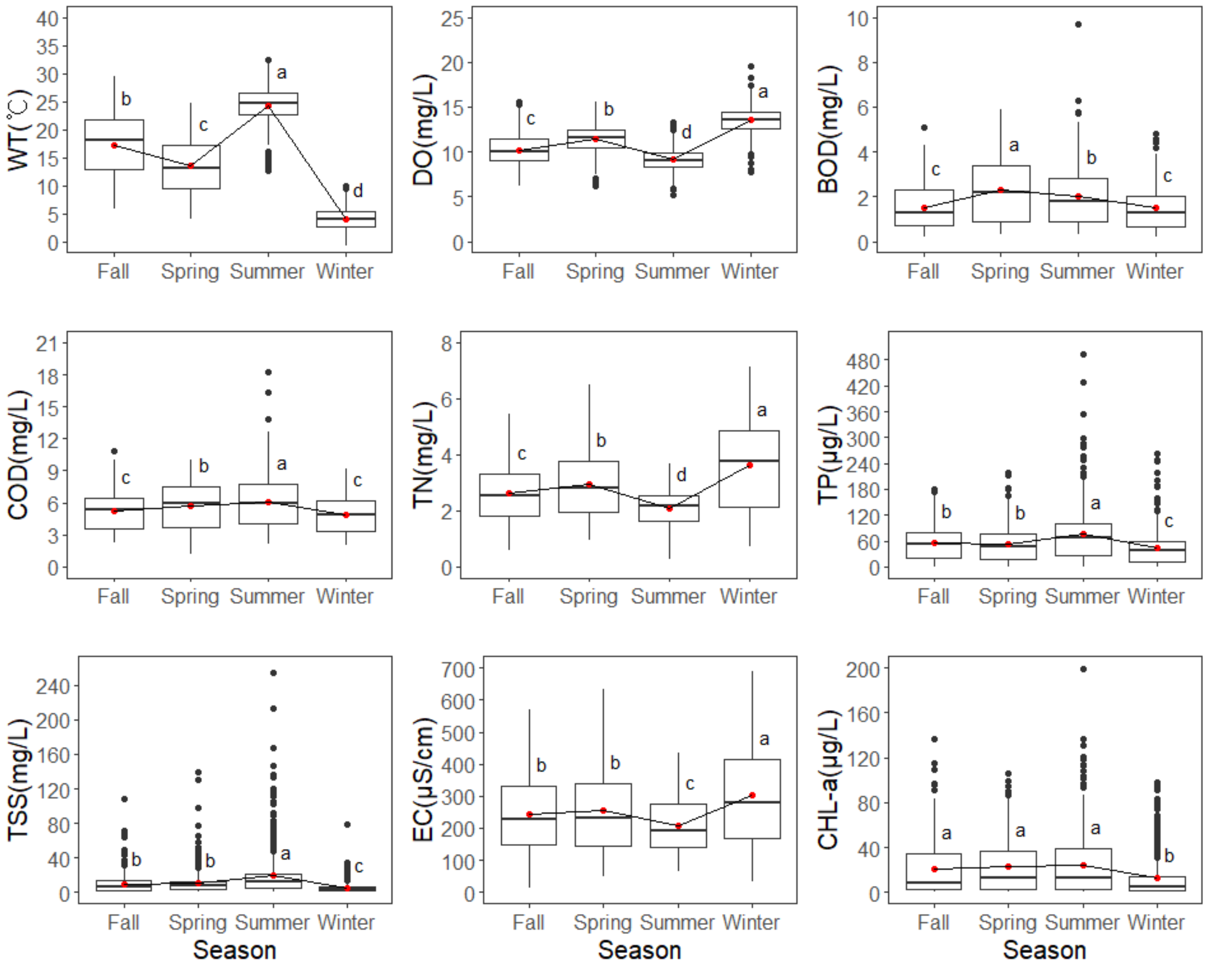

Figure S2. Seasonal variations of water quality parameters in the Geum River basin (WT: water temperature, DO: dissolved oxygen, BOD: biological oxygen demand, COD: chemical oxygen demand, TP: total phosphorus, TN: total nitrogen TSS: total suspended solids, EC: electrical conductivity and CHL-a: chlorophyll-a, Spring: Mar-May, Summer: Jun-Aug, Fall: Sep-Nov, Winter: Dec-Feb, the red dots indicate the mean value)

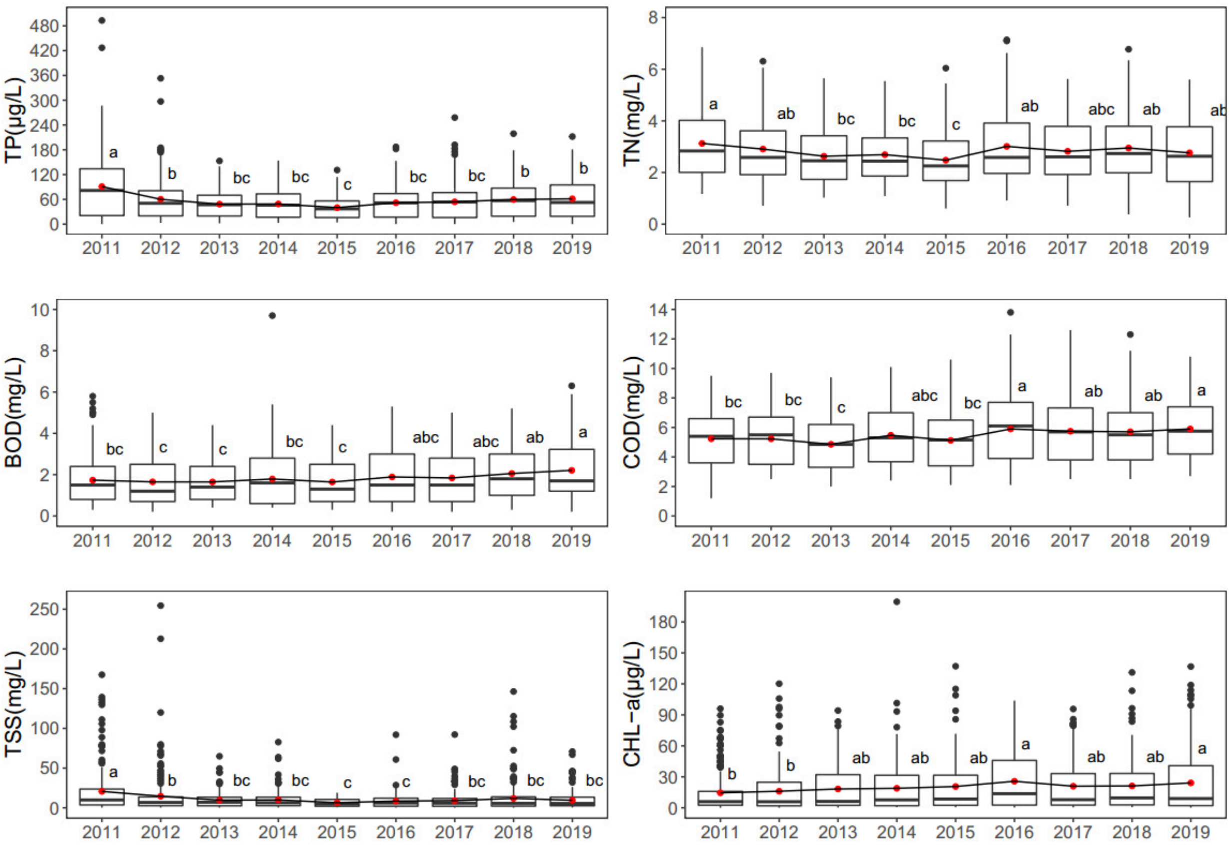

Figure S3. Yearly variations of water quality variables in the Geum River basin (TP: total phosphorus, TN: total nitrogen, BOD: biological oxygen demand, COD: chemical oxygen demand, TSS: total suspended solids, and CHL-a: chlorophyll-a, the red dots indicate the mean value)

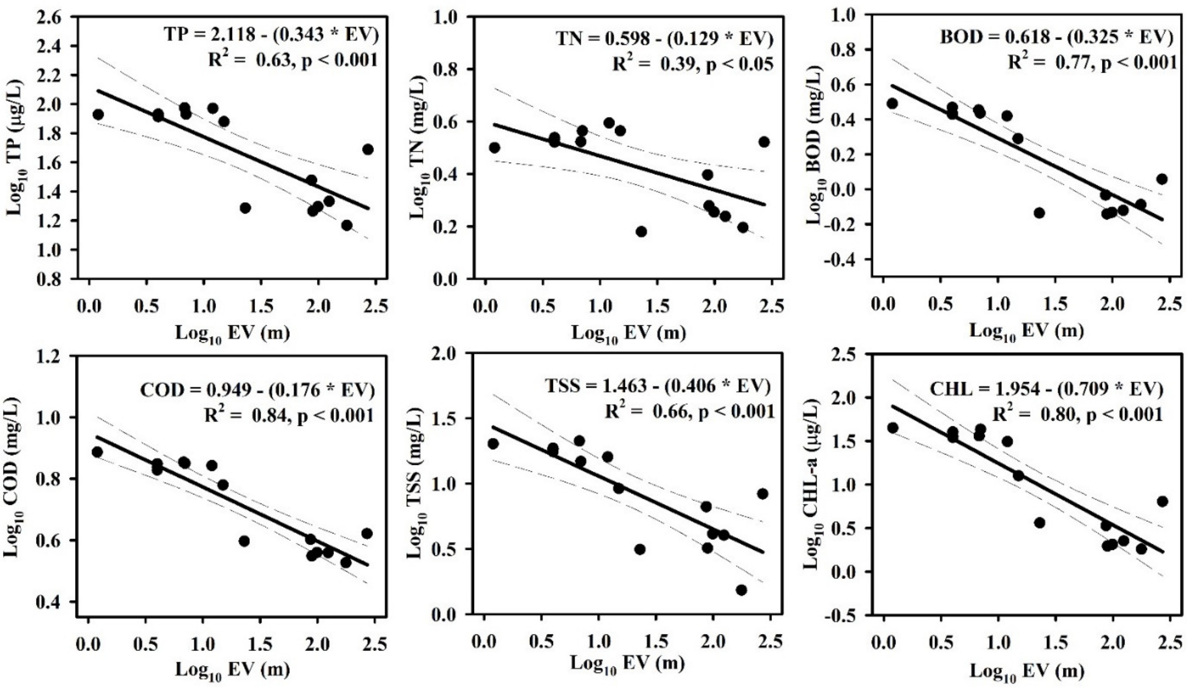

Figure S4. Relations of nutrients (TP: total phosphorus, TN: total nitrogen), organic matters (BOD: biological oxygen demand, COD: chemical oxygen demand), total suspended solids (TSS) and chlorophyll-a with elevation (EV) in the Geum River basin

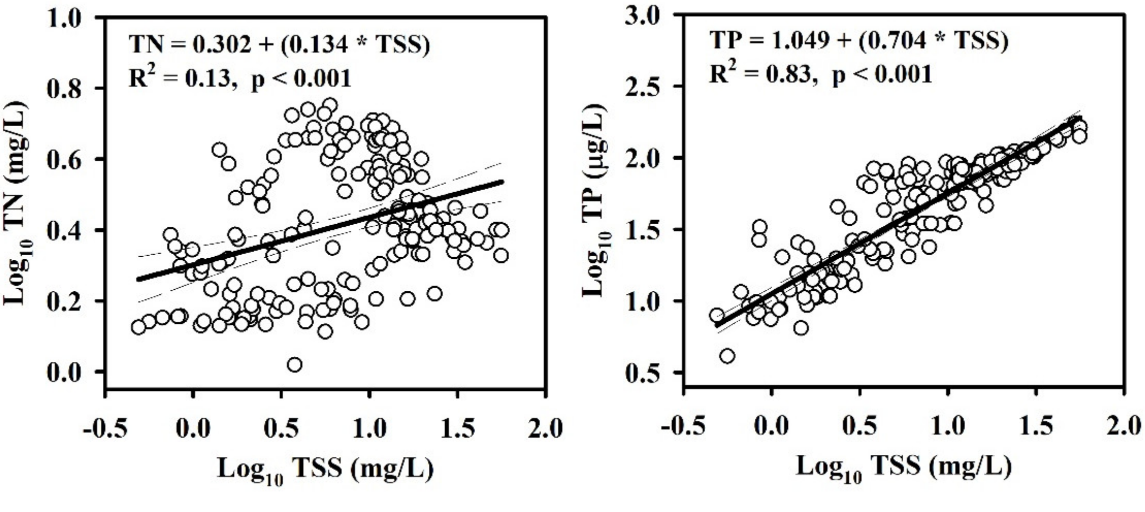

Figure S5. Relations of suspended solids with nutrients (TP: total phosphorus, TN: total nitrogen)

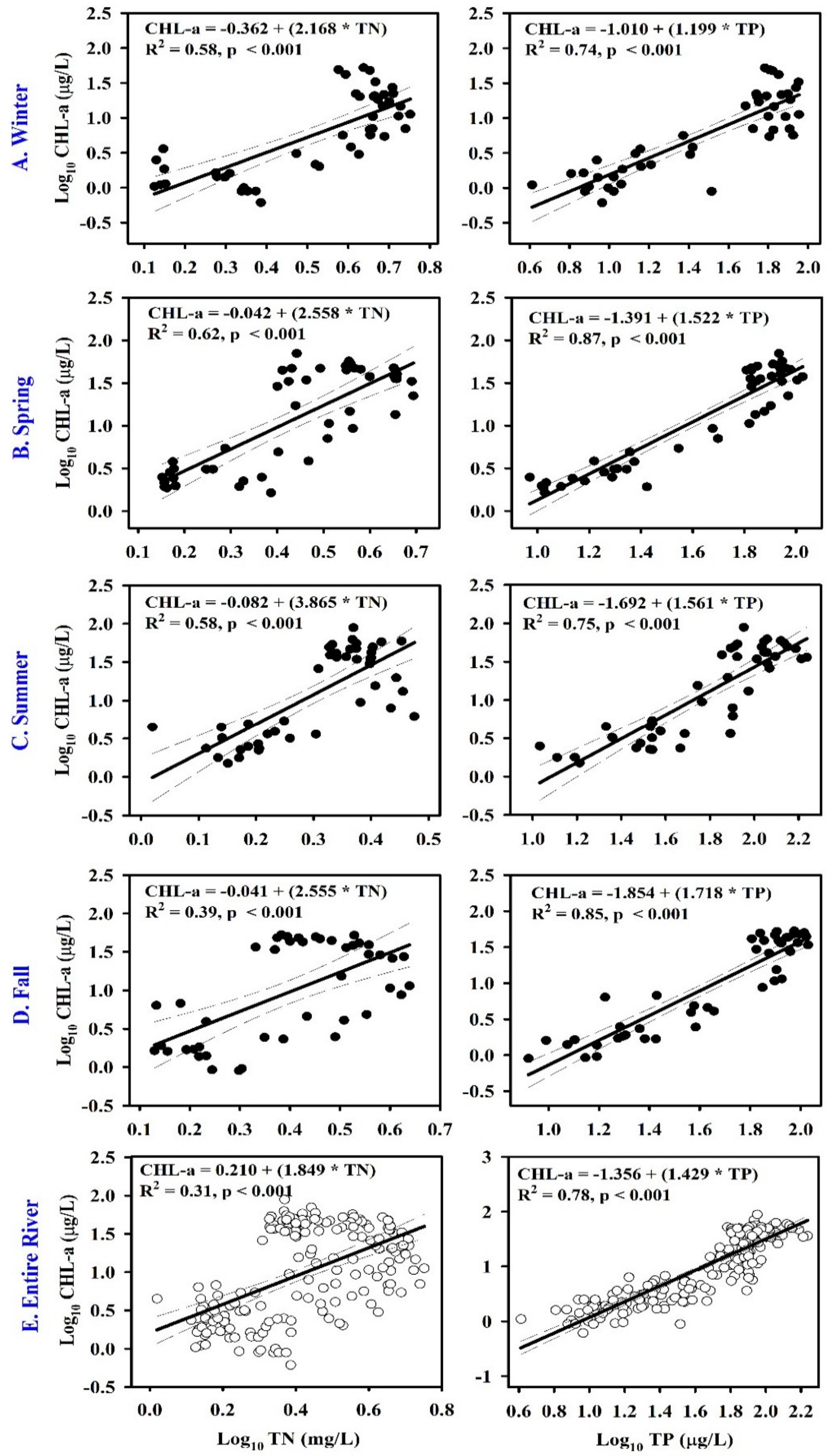

Figure S6. Relations of algal chlorophyll (CHL-a) with total nitrogen (TN) and total phosphorus (TP) in the Geum River (Spring: Mar-May, Summer: Jun-Aug, Fall: Sep-Nov, Winter: Dec-Feb)

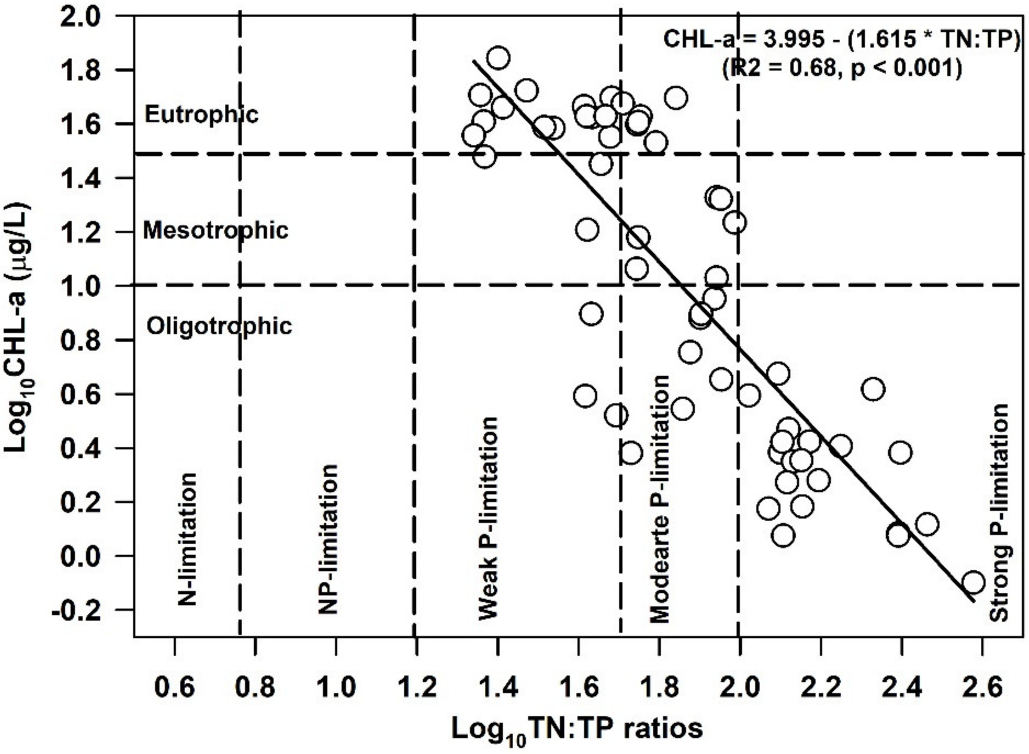

Figure S7. Nutrient limitation status determination based on the empirical relationship of algal chlorophyll (CHL-a) with TN:TP ratios.

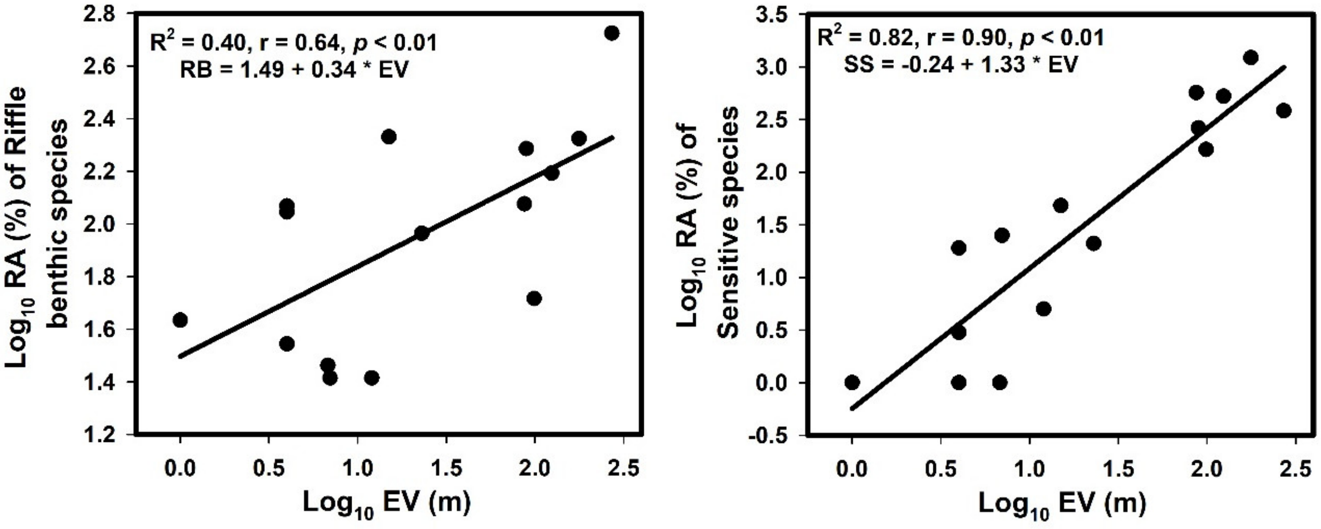

Figure S8. Relations of riffle benthic and sensitive fish species with elevation.

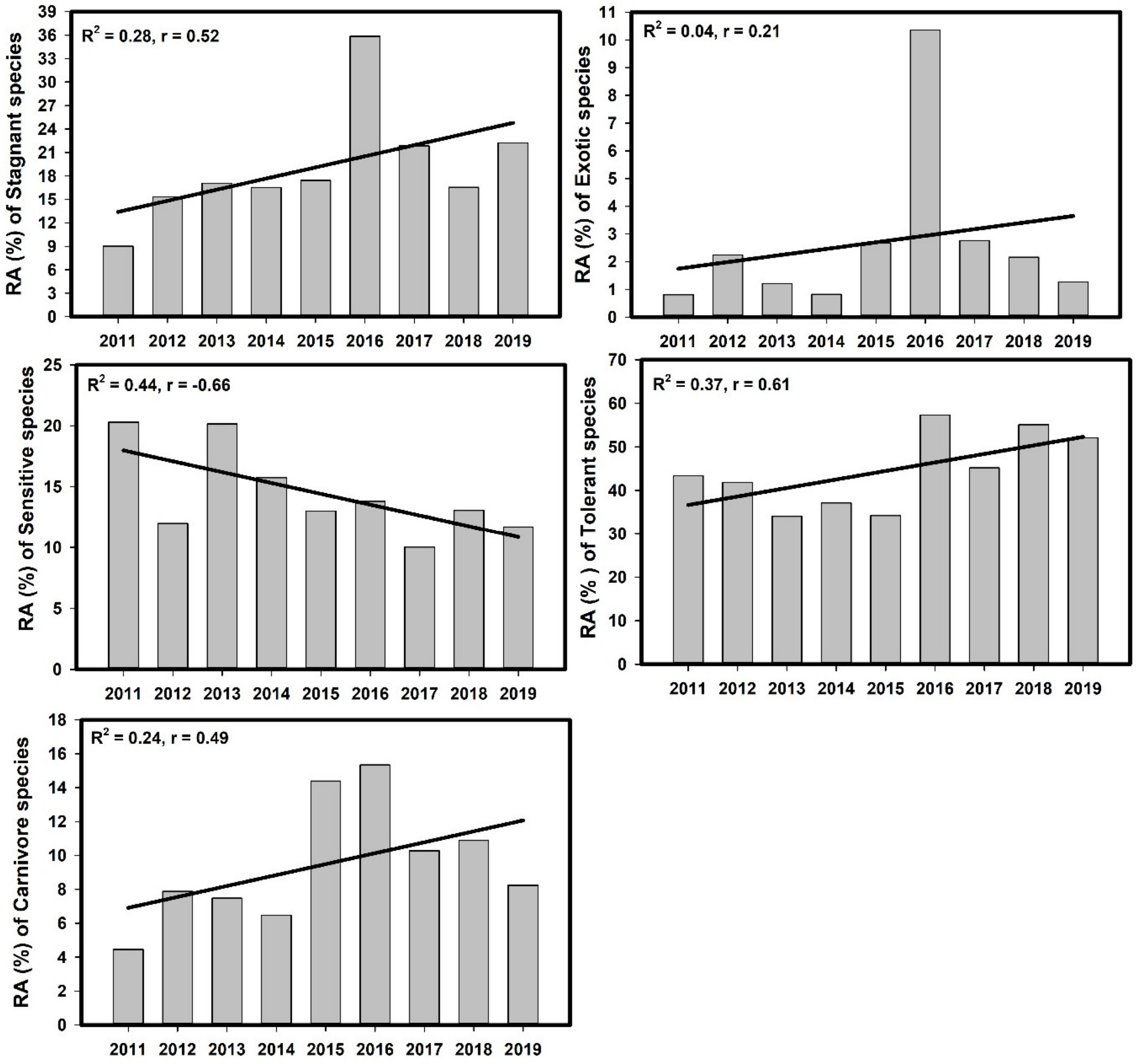

Figure S9. Yearly variations of stagnant, exotic, sensitive, tolerant, and carnivore fish species in the Geum River (the black line indicates the regression line)

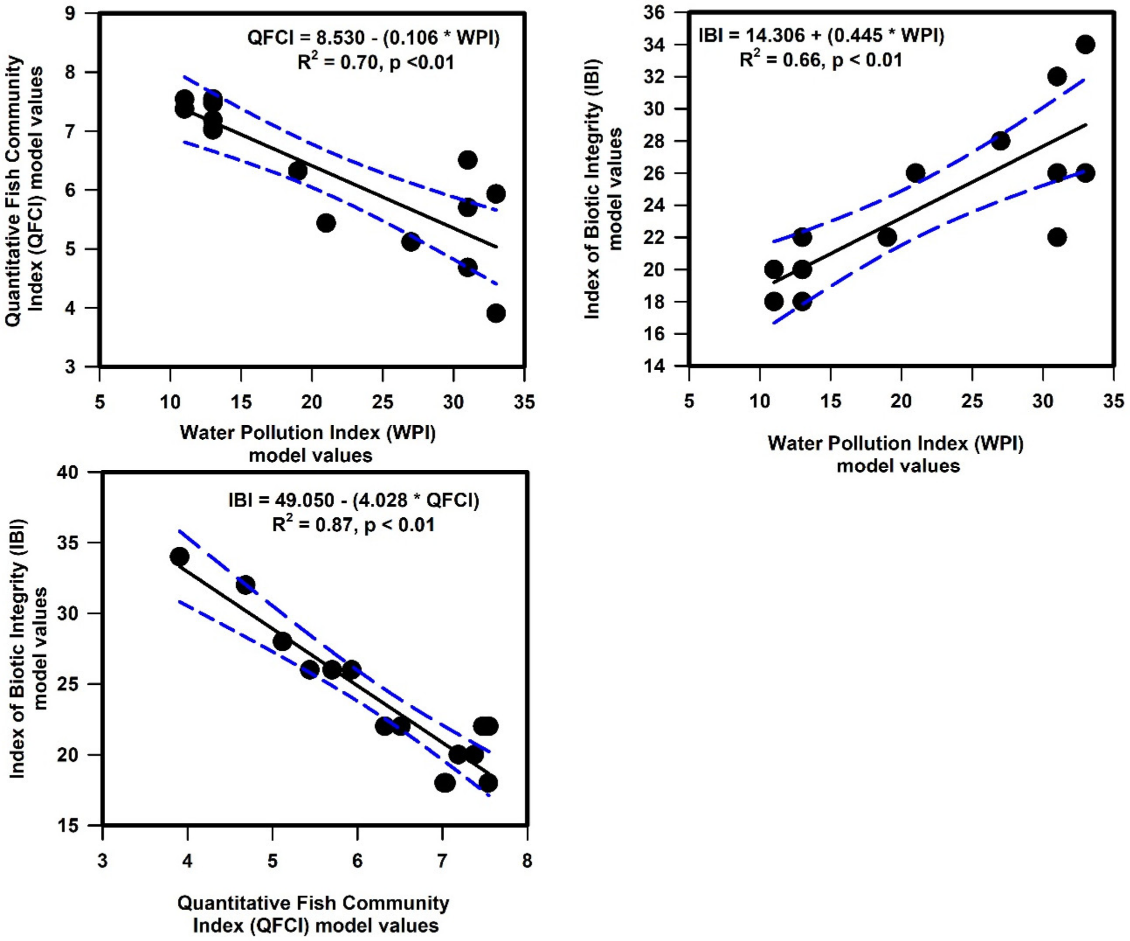

Figure S10. Relations among Water Pollution Index (WPI), Index of Biotic Integrity (IBI) and Quantitative Fish Community Index (QFCI) model values.

Table S1. Mann-Kendall trend analysis of water quality parameters in the Geum River basin. (WT: water temperature, EC: electrical conductivity, TSS: total suspended solids, BOD: biological oxygen demand, COD: chemical oxygen demand, TP: total phosphorus, TN: total nitrogen, CHL-a: chlorophyll-a)

Water Quality parameters	S value	P-value	Slope	Intercept	Trend	Sign
WT	10	0.17	0.09	14.31	No trend	↔
EC	20	0.02	11.15	196.87	Increasing trend	↑
TSS	-12	0.12	-0.92	15.75	No trend	↔
BOD	20	0.02	0.06	1.52	Increasing trend	↑
COD	18	0.03	0.10	4.93	Increasing trend	↑
TP	4	0.37	-1.75	65.99	No trend	↔
TN	-4	0.37	-0.01	2.87	No trend	↔
TN:TP	2	0.45	-0.05	93.33	No trend	↔
CHL-a	30	0.00	1.09	14.53	Increasing trend	↑

Table S2. Fish fauna and guild composition in the Geum River watershed. (Tol.G.: tolerance guild, Tro. G.: trophic guild, Hab. G.: habitat guild, RA: relative abundance, TNI: total number of individuals, TRA: total relative abundance, TNS: total number of species, ¥: exotic species, *: endangered species, SS: sensitive species, IS: intermediate species, TS: tolerant species, O: omnivores, I: insectivores, C: carnivores, H: herbivores, RB: riffle benthic species)

**Species**	Type of Fish Guild			Relative abundance of fish species (RA, %)															TNI	TRA(%)
	Tol. G.	Tro. G.	Hab. G.	S01	S02	S03	S04	S05	S06	S07	S08	S09	S10	S11	S12	S13	S14	S15		
Zacco platypus	TS	O		44.49	13.22	27.45	39.66	35.70	32.42	14.03	39.03	29.77	7.40	30.08	4.72	3.36	5.13	5.79	5696	25.62
Pseudogobio esocinus	IS	I		4.00	0.60	0.79	6.23	2.78	3.55	7.84	9.46	15.96	10.99	9.08	9.78	13.02	9.09	8.15	1398	6.29
Zacco koreanus	SS	I		7.59	23.13	12.64	0.06	12.52	2.18	0.78	0.63								1305	5.87
Pungtungia herzi	IS	I		6.81	8.67	14.35	10.31	7.04	3.28	7.45	0.91	0.08	0.24						1113	5.01
Hemibarbus labeo	TS	I		1.22	0.00	1.45	1.22	0.52	1.47	7.52	8.48	11.41	14.41	5.66	6.35	6.58	5.59	9.66	956	4.30
Acheilognathus lanceolata	IS	O			0.12	2.04	5.47	2.70	2.07	27.43	7.85	1.68	2.39	6.70	2.56	3.36			900	4.05
Acheilognathus yamatsutae	IS	O		0.04	3.64	5.46	8.10	13.22	12.61	1.80	1.26			0.30					894	4.02
Microphysogobio jeoni	IS	I										0.08	5.73	4.62	36.11	9.26	3.96	5.58	867	3.90
Rhinogobius brunneus	IS	I	RB	10.07	0.08	0.20	1.05	0.61	5.51	3.21	8.83	1.12	1.59	7.37	6.00	3.89	0.70	1.93	829	3.73
Opsariichthys uncirostris amurensis	TS	C		3.43		0.53	0.76	0.17	2.46		2.73	6.30	2.79	3.35	12.29	22.01	12.82	7.73	818	3.68
Acheilognathus koreensis	IS	O		0.16	21.97	5.07	0.82	3.09	0.55										726	3.27
Squalidus japonicus coreanus	TS	O		0.04					0.16	0.16	0.84	2.15	11.31	8.71	8.10	8.86	16.78	11.16	633	2.85
Coreoleuciscus splendidus	SS	I	RB	4.24	6.83	8.03	1.69	1.17	1.31	0.31	0.63								490	2.20
Carassius auratus	TS	O		0.04		0.07	2.62	0.13	0.38	7.76	0.70	11.97	5.73	1.12	0.41	1.74	2.80	4.08	454	2.04
*Pseudopungtungia nigra**	SS	I			7.67	5.40	0.35	2.04	3.00										382	1.72
Hemibarbus longirostris	IS	I		4.00	0.80	2.24	2.74	1.13	2.40	2.82	1.40	0.08	0.80	0.97	0.29	1.61		0.21	367	1.65
Squaliobarbus curriculus	IS	O							0.11	0.63	0.21	0.64	2.79	2.76	3.61	4.43	24.24	15.24	363	1.63

Micropterus salmoides ¥	TS	C		1.31		1.51	1.81	0.04	0.55	1.18	1.33	1.36	4.94	4.91	0.76	2.55	3.73	7.08	357	1.61
Sarcocheilichthys variegatus	SS	I			0.08	2.37	4.31	2.91	5.51	0.00	0.56	0.08		0.30					293	1.32
Squalidus chankaensis tsuchigae	IS	O		0.08				1.04	6.33	0.39	0.42	0.72	3.26	1.79	1.40	5.37		0.21	292	1.31
Microphysogobio yaluensis	IS	O	RB	5.10	0.04	1.25	0.29	1.87	3.06	0.08	0.77	0.80	0.08		0.12				274	1.23
Zacco temminckii	SS	I		0.69	6.47	2.04		1.43	0.60										254	1.14
Hemiculter eigenmanni	TS	O							3.71	0.08	0.00	0.40	2.87	3.28	1.63	6.31	0.23	0.86	234	1.05
Odontobutis interrupta	IS	C		0.12		0.59	3.44	1.61	0.44	3.61	2.94	1.60	0.24	0.52					234	1.05
Tridentiger brevispinis	IS	I	RB	0.08				0.13	0.55	3.61	3.99	0.16	0.32	1.34	0.35	0.81	6.06	7.30	214	0.96
Erythroculter erythropterus	TS	C								0.24	0.28	1.68	0.80	0.60	3.84	3.89	4.43	10.73	210	0.94
Coreoperca herzi	SS	C		0.53	3.00	1.71	1.34	1.91	0.93	0.08									199	0.89
Lepomis macrochirus ¥	TS	I					0.06	0.26	0.11	0.94	0.28	0.32	12.10			0.67			186	0.84
Pseudorasbora parva	TS	O					0.29	0.04	0.00	0.71	0.42	8.06	0.32	1.19	0.41	0.81	0.23	0.64	159	0.72
Odontobutis platycephala	SS	C		0.57	0.92	0.92	0.82	1.26	0.44	0.47	0.21			0.07					112	0.50
Sarcocheilichthys nigripinnis	IS	I			0.04	0.39	0.99	1.78	0.33	0.24	0.63			0.89			0.93		99	0.45
Iksookimia koreensis	IS	I	RB	1.96	1.04	0.39	0.00	0.13	0.11	0.00				0.00					85	0.38
Acheilognathus rhombeus	IS	O				0.39	1.05	0.04	2.46	0.00	0.56		0.16	0.15					82	0.37
Rhinogobius giurinus	TS	O							0.11	0.24	0.56		0.56	0.97	0.93		1.63	0.64	59	0.27
Acanthorhodeus chankaensis	IS	O							0.16	2.51	0.00	0.48	0.48	0.52		0.40	0.23		58	0.26
Rhodeus uyekii	IS	C			0.64	1.25	0.29	0.57					0.08						54	0.24
Pseudobagrus koreanus	SS	I	RB	0.16	0.32	0.46		1.00			0.77								53	0.24
Siniperca scherzeri	SS	C		0.73	0.08	0.20	0.99	0.09	0.27	0.00	0.21	0.08	0.08	0.07					53	0.24
Rhodeus notatus	IS	O					0.93	0.26		1.18	0.07		0.56	0.37					50	0.22
Cyprinus carpio	TS	O					0.17			0.55	0.35	0.48	1.59	0.37		0.13		0.21	48	0.22

Acheilognathus macropterus	IS	O					0.12		0.05	0.00	0.35	0.64	1.51	0.45	0.00	0.00	0.70	0.21	45	0.20
Gnathopogon strigatus	IS	I							0.11	0.24	1.47	0.80	0.40						41	0.18
Rhynchocypris oxycephalus	SS	I		0.73								0.08	1.67						40	0.18
Squalidus gracilis majimae	SS	I		0.29	0.08	0.13		0.04	0.11			0.16	0.16	0.97		0.40			34	0.15
Misgurnus anguillicaudatus	TS	O		0.41			0.06			1.33	0.07	0.16			0.06	0.13			33	0.15
Pseudobagrus fulvidraco	TS	I					0.82	0.09	0.55	0.08		0.08				0.40	0.23	0.21	33	0.15
Misgurnus mizolepis	TS	O		0.16	0.12		0.06	0.09		0.47	0.21	0.24	0.16		0.06				25	0.11
Plecoglossus altivelis	IS	H		0.90															22	0.10
Abbottina rivularis	TS	O										0.16	0.48	0.45	0.23				18	0.08
*Gobiobotia brevibarba**	SS	I	RB		0.08	0.53		0.22											15	0.07
Carassius cuvieri ¥	TS	O					0.29				0.14	0.08	0.24						11	0.05
Macropodus ocellatus	TS	I					0.58												10	0.04
Oryzias sinensis	TS	O			0.16													1.29	10	0.04
Rhodeus ocellatus	IS	O											0.72						9	0.04
Channa argus	TS	C				0.07	0.17				0.07	0.08							6	0.03
Cobitis choii	SS	I									0.35								5	0.02
Silurus asotus	TS	C					0.06	0.09		0.08							0.23		5	0.02
Liobagrus obesus	SS	I	RB					0.17											4	0.02
Tridentiger obscurus	TS	I																0.86	4	0.02
Leiocassis ussuriensis	IS	I			0.04			0.09											3	0.01
Oryzias latipes	TS	O			0.12														3	0.01
Hypomesus nipponensis	IS	I										0.080		0.074					2	0.009
Liobagrus mediadiposalis	SS	I	RB	0.041	0.040														2	0.009
Mugil cephalus	TS	H															0.233	0.215	2	0.009

Cobitis nalbanti	IS	I							0.055										1	0.004
*Gobiobotia macrocephala**	SS	I	RB			0.066													1	0.004
*Gobiobotia nakdongensis**	SS	I	RB										0.080						1	0.004
TNS				30	28	31	36	39	38	34	38	36	37	32	22	23	21	23		
TNI				2452	2503	1519	1717	2300	1832	1276	1427	1253	1256	1343	1717	745	429	466	22,235	

Table S3. Sites-based Chemical health assessment (CHA) based on multi-metric water pollution index (WPI) in the Geum River basin. (EX: excellent, G: good, F: fair, P: poor, VP: very poor.

Category	Model Metric	Scoring Criteria			Geum River														
		5	3	1	S01	S02	S03	S04	S05	S06	S07	S08	S09	S10	S11	S12	S13	S14	S15
Nutrient regime	M_1: total nitrogen (mgL^{-1})	<1.5	1.5-3.0	>3	3.33 (1)	1.57 (3)	1.73 (3)	1.80 (3)	2.49 (3)	1.90 (3)	1.51 (3)	3.67 (1)	3.93 (1)	3.67 (1)	3.45 (1)	3.40 (1)	3.32 (1)	3.34 (1)	3.16 (1)
	M_2: total phosphorus (μgL^{-1})	<30	30-100	>100	48.57 (3)	15.44 (5)	21.48 (5)	19.75 (5)	29.98 (5)	18.37 (5)	19.32 (5)	75.67 (3)	93.44 (3)	85.14 (3)	84.77 (3)	85.18 (3)	81.78 (3)	94.02 (3)	84.68 (3)
	M_3: TN:TP ratio	>50	20-50	<20	108.1 (5)	151.2 (5)	145.5 (5)	154.3 (5)	135.1 (5)	183.1 (5)	114.5 (5)	58.2 (5)	50.6 (5)	50.9 (5)	55.1 (5)	51.4 (5)	52.2 (5)	41.5 (3)	45.2 (3)
Organic matter	M_4: biological oxygen demand (mgL^{-1})	<1	1-2.5	>2.5	1.14 (3)	0.82 (5)	0.76 (5)	0.74 (5)	0.93 (5)	0.72 (5)	0.73 (5)	1.95 (3)	2.62 (1)	2.73 (1)	2.71 (1)	2.94 (1)	2.68 (1)	2.84 (1)	3.09 (1)
Ionic contents and solids	M_5: total suspended solid (mgL^{-1})	<4	4-10	>10	8.32 (3)	1.53 (5)	4.02 (3)	4.11 (3)	6.65 (3)	3.20 (5)	3.13 (5)	9.14 (3)	16.00 (1)	14.79 (1)	18.37 (1)	17.55 (1)	18.66 (1)	21.18 (1)	20.20 (1)
	M_6: electrical conductivity (μScm^{-1})	<180	180-300	>300	184.7 (3)	107.8 (5)	126.0 (5)	140.3 (5)	180.6 (3)	158.1 (5)	153.3 (5)	289.6 (3)	382.9 (1)	370.5 (1)	346.9 (1)	350.4 (1)	334.1 (1)	336.8 (1)	321.6 (1)
Primary production indicator	M_7: chlorophyll (μgl^{-1})	<3	3-10	>10	6.37 (3)	1.81 (5)	2.25 (5)	2.05 (5)	3.36 (3)	1.96 (5)	3.63 (3)	12.61 (1)	31.14 (1)	43.07 (1)	35.73 (1)	40.13 (1)	34.76 (1)	36.18 (1)	44.86 (1)
**CHA Scores**					**21**	**33**	**31**	**31**	**27**	**33**	**31**	**19**	**13**	**13**	**13**	**13**	**13**	**11**	**11**
**Chemical Status of the River**					**F**	**EX**	**EX**	**EX**	**G**	**EX**	**EX**	**F**	**P**	**P**	**P**	**P**	**P**	**VP**	**VP**

Table S4. Yearly Chemical health assessment (CHA) based on multi-metric water pollution index (WPI) in the Geum River basin. (F: fair, P: poor).

**Category**	**Model Metric**	**Scoring Criteria**			**Geum River**								
		**5**	**3**	**1**	**2011**	**2012**	**2013**	**2014**	**2015**	**2016**	**2017**	**2018**	**2019**
**Nutrient regime**	M_1: total nitrogen (mgL^{-1})	<1.5	1.5-3.0	>3	3.13 (1)	2.91 (3)	2.63 (3)	2.69 (3)	2.48 (3)	3.01 (1)	2.82 (3)	2.95 (3)	2.76 (3)
	M_2: total phosphorus (μgL^{-1})	<30	30-100	>100	90.89 (3)	60.11 (3)	48.35 (3)	48.78 (3)	39.99 (3)	52.08 (3)	54.07 (3)	59.58 (3)	61.34 (3)
	M_3: TN:TP ratio	>50	20-50	<20	83.6 (5)	91.4 (5)	96.1 (5)	97.3 (5)	92.5 (5)	96.6 (5)	118.1 (5)	85.4 (5)	76.4 (5)
**Organic matter**	M_4: biological oxygen demand (mgL^{-1})	<1	1-2.5	>2.5	1.73 (3)	1.65 (3)	1.65 (3)	1.79 (3)	1.64 (3)	1.89 (3)	1.84 (3)	2.06 (3)	2.21 (3)
**Ionic contents and solids**	M_5: total suspended solid (mgL^{-1})	<4	4-10	>10	20.68 (1)	14.60 (1)	9.65 (3)	9.92 (3)	6.39 (3)	8.47 (3)	9.03 (3)	11.88 (1)	9.51 (3)
	M_6: electrical conductivity (μScm^{-1})	<180	180-300	>300	207.5 (3)	203.3 (3)	214.9 (3)	243.8 (3)	267.6 (3)	299.7 (3)	292.4 (3)	279.8 (3)	264.7 (3)
**Primary production indicator**	M_7: chlorophyll (μgl^{-1})	<3	3-10	>10	14.53 (1)	16.05 (1)	18.24 (1)	18.90 (1)	20.63 (1)	25.72 (1)	20.93 (1)	21.20 (1)	24.12 (1)
**CHA Scores**					**17**	**19**	**21**	**21**	**21**	**19**	**21**	**19**	**21**
**Chemical Status of the River**					**P**	**F**	**F**	**F**	**F**	**F**	**F**	**F**	**F**

Table S5. Sites-based Biological health assessment (BHA), based on the index of biotic integrity (IBI_{KR}) using fish assemblages in the Geum River Basin. (F: fair, G: good, P: poor)

**Model Category**	**Model Metric**	**Scoring Criteria**			**Geum River**														
		**5**	**3**	**1**	**S01**	**S02**	**S03**	**S04**	**S05**	**S06**	**S07**	**S08**	**S09**	**S10**	**S11**	**S12**	**S13**	**S14**	**S15**
**Species richness and composition**	M_1: total number of native fish species	Expectations of M_1-M_3 vary with stream size and region			29<br>(5)	28<br>(5)	31<br>(5)	33<br>(5)	37<br>(5)	36<br>(5)	32<br>(5)	35<br>(5)	33<br>(5)	34<br>(5)	31<br>(5)	21<br>(5)	21<br>(5)	20<br>(5)	22<br>(5)
	M_2: number of riffle benthic species				7<br>(5)	7<br>(5)	7<br>(5)	3<br>(3)	7<br>(5)	5<br>(3)	4<br>(3)	5<br>(3)	3<br>(1)	4<br>(3)	2<br>(1)	3<br>(1)	2<br>(1)	2<br>(1)	2<br>(1)
	M_3: number of sensitive species				10<br>(5)	12<br>(5)	12<br>(5)	7<br>(5)	12<br>(5)	9<br>(5)	4<br>(1)	7<br>(3)	4<br>(1)	4<br>(1)	4<br>(1)	0<br>(1)	1<br>(1)	0<br>(1)	0<br>(1)
	M_4: proportion of individuals as tolerant species	<5%	5-20%	>20%	51.10<br>(1)	13.62<br>(3)	31.28<br>(1)	48.63<br>(1)	31.73<br>(1)	41.92<br>(1)	35.29<br>(1)	55.50<br>(1)	74.70<br>(1)	65.68<br>(1)	60.69<br>(1)	39.78<br>(1)	57.45<br>(1)	54.08<br>(1)	61.69<br>(1)
**Trophic composition**	M_5: proportion of individuals as omnivore species	<20%	20-45%	>45%	50.53<br>(1)	39.39<br>(3)	42.61<br>(3)	60.22<br>(1)	58.87<br>(1)	64.19<br>(1)	59.37<br>(1)	53.82<br>(1)	58.42<br>(1)	42.68<br>(3)	59.20<br>(1)	24.23<br>(3)	34.90<br>(3)	51.98<br>(1)	40.34<br>(3)
	M_6: proportion of individuals as native insectivore species	>45%	20-45%	<20%	41.88<br>(3)	56.61<br>(5)	51.82<br>(5)	30.36<br>(3)	35.79<br>(3)	30.66<br>(3)	34.44<br>(3)	38.23<br>(3)	30.26<br>(3)	41.39<br>(3)	31.27<br>(3)	58.88<br>(5)	36.22<br>(3)	26.57<br>(3)	33.91<br>(3)

**Fish abundance and condition**	M_7: total number of native individuals	Expectations of M_7 vary with stream size and region			2420 (5)	2503 (5)	1519 (5)	1680 (5)	2293 (5)	1820 (5)	1249 (5)	1427 (5)	1231 (5)	1039 (5)	1277 (5)	1704 (5)	721 (5)	413 (5)	433 (5)
	M_8: percent of individuals with anomalies	0%	0-1%	>1%	>1 (1)	<1 (3)	<1 (3)	<1 (3)	<1 (3)	<1 (3)	<1 (3)	>1 (1)	>1 (1)	>1 (1)	>1 (1)	>1 (1)	>1 (1)	>1 (1)	>1 (1)
**Overall IBI Scores**					**26**	**34**	**32**	**26**	**28**	**26**	**22**	**22**	**18**	**22**	**18**	**22**	**20**	**18**	**20**
**Biological Health Status of the River**					**F**	**G**	**G**	**F**	**G**	**F**	**F**	**F**	**P**	**F**	**P**	**F**	**F**	**P**	**F**

Table S6. Yearly biological health assessment (BHA), based on the index of biotic integrity (IBI_{KR}) using fish assemblages in the Geum River Basin. (F: fair, G: Good)

**Model Category**	**Model Metric**	**Scoring Criteria**			**Geum River**								
		**5**	**3**	**1**	**2011**	**2012**	**2013**	**2014**	**2015**	**2016**	**2017**	**2018**	**2019**
**Species richness and composition**	M_1: total number of native fish species	Expectations of M_1-M_3 vary with stream size and region			46 (5)	44 (5)	42 (5)	47 (5)	46 (5)	42 (5)	48 (5)	44 (5)	50 (5)
	M_2: number of riffle benthic species				6 (3)	7 (5)	7 (5)	8 (5)	9 (5)	8 (5)	6 (5)	7 (5)	8 (5)
	M_3: number of sensitive species				11 (5)	11 (5)	10 (5)	12 (5)	12 (5)	10 (5)	9 (5)	9 (5)	13 (5)
	M_4: proportion of individuals as tolerant species	<5%	5-20%	>20%	43.35 (1)	41.81 (1)	34.05 (1)	37.14 (1)	34.26 (1)	57.34 (1)	45.19 (1)	55.12 (1)	52.07 (1)
**Trophic composition**	M_5: proportion of individuals as omnivore species	<20%	20-45%	>45%	56.43 (1)	48.14 (1)	46.26 (1)	48.99 (1)	38.82 (1)	46.58 (1)	55.17 (1)	56.85 (1)	52.19 (1)
	M_6: proportion of individuals as native insectivore species	>45%	20-45%	<20%	39.12 (3)	43.99 (3)	46.11 (5)	44.48 (3)	46.74 (5)	38.09 (3)	34.04 (3)	31.63 (3)	38.69 (3)
**Fish abundance and condition**	M_7: total number of native individuals	Expectations of M_7 vary with stream size and region			3170 (5)	2483 (5)	2530 (5)	2420 (5)	2003 (5)	1920 (5)	1940 (5)	2268 (5)	2947 (5)
	M_8: percent of individuals with anomalies	0%	0-1%	>1%	>1 (1)	>1 (1)	>1 (1)	>1 (1)	>1 (1)	>1 (1)	>1 (1)	>1 (1)	>1 (1)
**Overall IBI Scores**					**24**	**26**	**28**	**26**	**28**	**26**	**26**	**26**	**26**
**Biological Health Status of the River**					**F**	**F**	**G**	**F**	**G**	**F**	**F**	**F**	**F**

Table S7. Canonical correspondence analysis of trophic and tolerance guilds with water quality variables and land-use pattern and elevation. IS: intermediate species, SS: sensitive species, TS: tolerant species, C: carnivore, I: Insectivores, O: omnivores, AG: agricultural land-use coverage, WT: water temperature, BOD: biological oxygen demand, COD: chemical oxygen demand, TSS: total suspended solids, TP: total phosphorus, TN: total nitrogen, CHL-a: chlorophyll-a and EC: electrical conductivity (*p<0.05)

Variables	Parameters	Axis 1	Axis 2
Response variables	IS	-0.13	0.24
	SS	3.82	0.52
	TS	-0.72	-0.32
	C	-1.23	2.84
	I	0.35	0.74
	O	0.01	-1.24
Environmental Variables	% AG	-0.03	0.24
	% Forest	0.56	-0.27
	% Built-up	-0.61	0.12
	Elevation (m)	0.72	-0.10
	WT	-0.23	0.39
	BOD	-0.74	0.39
	COD	-0.78	0.32
	TSS	-0.76	0.37
	TN	-0.68	0.08
	TP	-0.76	0.25
	TN:TP	0.75	-0.30
	CHL-a	-0.72	0.41
	EC	-0.79	0.19
% of variance		72*	14.6
Cumulative %		72	86.6
